# UKB.COVID19: an R package for UK Biobank COVID-19 data processing and analysis

**DOI:** 10.12688/f1000research.55370.3

**Published:** 2024-07-26

**Authors:** Longfei Wang, Victoria E Jackson, Liam G Fearnley, Melanie Bahlo

**Affiliations:** 1 Department of Medical Biology, The University of Melbourne, Parkville, VIC, 3010, Australia; 2 Population Health and Immunity Division, The Walter and Eliza Hall Institute of Medical Research, Parkville, VIC, 3052, Australia

**Keywords:** R package, UK Biobank, COVID-19, GWAS, risk factors

## Abstract

COVID-19 caused by SARS-CoV-2 has resulted in a global pandemic with a rapidly developing global health and economic crisis. Variations in the disease have been observed and have been associated with the genomic sequence of either the human host or the pathogen. Worldwide scientists scrambled initially to recruit patient cohorts to try and identify risk factors. A resource that presented itself early on was the UK Biobank (UKBB), which is investigating the respective contributions of genetic predisposition and environmental exposure to the development of disease. To enable COVID-19 studies, UKBB is now receiving COVID-19 test data for their participants every two weeks. In addition, UKBB is delivering more frequent updates of death and hospital inpatient data (including critical care admissions) on the UKBB Data Portal. This frequently changing dataset requires a tool that can rapidly process and analyse up-to-date data. We developed an R package specifically for the UKBB COVID-19 data, which summarises COVID-19 test results, performs association tests between COVID-19 susceptibility/severity and potential risk factors such as age, sex, blood type, comorbidities and generates input files for genome-wide association studies (GWAS). By applying the R package to data released in April 2021, we found that age, body mass index, socioeconomic status and smoking are positively associated with COVID-19 susceptibility, severity, and mortality. Males are at a higher risk of COVID-19 infection than females. People staying in aged care homes have a higher chance of being exposed to SARS-CoV-2. By performing GWAS, we replicated the 3p21.31 genetic finding for COVID-19 susceptibility and severity. The ability to iteratively perform such analyses is highly relevant since the UKBB data is updated frequently. As a caveat, users must arrange their own access to the UKBB data to use the R package.

## Introduction

The ongoing global pandemic of coronavirus disease 2019 (COVID-19), caused by a novel coronavirus (severe acute respiratory syndrome coronavirus 2, SARS-CoV-2), has resulted in a rapidly developing global health and economic crisis. Most people with COVID-19 never develop symptoms or suffer mild symptoms. However, about 5% of cases are critical (defined as respiratory failure, septic shock, and/or multiorgan dysfunction or failure) ( Wu and McGoogan 2020), possibly leading to lethal lung damage and even death. These and other clinical observations led to the hypothesis that genetic factors in either or both the host and the pathogen could be responsible, at least in part, for this variation. Worldwide scientists scrambled initially to recruit patient cohorts to try and identify genetic risk factors.

UK Biobank (UKBB) (RRID: SCR_012815) is a long-term biobank study that recruited 500,000 volunteers aged between 40–69 years in 2006–2010 from across the UK. UKBB’s large-scale database is a global research resource accessible to approved researchers who are undertaking health-related research. All participants provided detailed information about their lifestyle, physical measures and had blood, urine and saliva samples collected. The samples of all participants have undergone SNP array typing and are now also undergoing whole-exome and whole-genome sequencing. UKBB has become a major contributor to the advancement of modern medicine and treatment, enabling a better understanding of a wide range of serious and life-threatening diseases.

Researchers can apply for access to the data and worldwide hundreds of researchers are using the UKBB data to carry out research on many different diseases. The UKBB has facilitated first-time analyses on traits such as brain imaging phenotypes ( Elliott *et al*., 2018).

The UK has been badly affected by COVID-19. As of 20 May 2021, there have been over 127,000 reported deaths in the UK, with an estimated 4.5 million infections. Worldwide there have now been more than 3 million reported deaths due to COVID-19, with continually increasing rates of infections in India and South America. The UKBB was an early, available population genetic resource that could be harnessed to better understand COVID-19 risk factors, and with its continuing evolution continues to serve as a powerful cohort to permit such studies.

UKBB has taken swift strides to help tackle the global pandemic by undertaking four major initiatives: serology study, COVID-19 repeat imaging study, coronavirus self-test antibody study and health data linkage. UKBB has been receiving COVID-19 test data for previous UKBB participants in England and has linked the test result data with health data. The test results data are being updated every two weeks. In addition, UKBB is making more frequent updates of death and hospital inpatient data (including critical care admissions) on the Data Portal. This rapidly changing dataset requires a tool that can process the up-to-date data as frequently as the data updates, in a standardised, reproducible, and somewhat automated manner to permit rapid re-analysis of the data and to also enable other researchers to use such a tool as a basis for their analyses.

Therefore, we developed an R package (version 4.0.5) UKB.COVID-19 which summarises COVID-19 test results, combines test results data with hospitalisation data and death register data, performs association tests between COVID-19 susceptibility/severity and potential risk factors (age, sex, blood type, socioeconomic status, comorbidities etc.) and generates input files for genome-wide association studies (GWAS). Ethics approval was granted through WEHI project 17/09LR by the WEHI’s Human Research Ethics Committee (HREC).

## Methods

### Implementation

UKB.COVID19 was built in R (version 4.0.5) and currently depends on the following R packages: *questionr, data.table, tidyverse, magrittr, here*, and *dplyr.* COVID-19 related data files from UKBB can be directly imported in the R package without any pre-processing.

### Operation

UKB.COVID19 is distributed as part of the CRAN R package repository and is compatible with Mac OS X, Windows, and major Linux operating systems. UKB.COVID19 is maintained at GitHub ( https://github.com/bahlolab/UKB.COVID19). The archived source code can be found in http://doi.org/10.5281/zenodo.5174381 ( Wang *et al*., 2021). All analyses are performed using R (version 4.0.5). All functions and descriptions are listed in Table 1.

**Table 1.** Description of R functions in the UKB.COVID19 R package.

Function	Description
*risk_factor*	Selects several potential non-genetic risk factors from the linked health data provided by UKBB and generates an output file including the selected risk factors for the downstream analyses. Automatically returns sex, age at birthday in 2020, socioeconomic status, self-reported ethnicity, most recently reported body mass index, most recently reported pack-years of smoking, whether they reside in aged care (based on hospital admissions data, and COVID-19 test data) and blood type. Function also allows users to specify fields of interest (field codes, provided by UK Biobank), and allows the user to specify more intuitive names for selected fields.
*makePhenotypes*	Summarises COVID-19 test results data, death register data and hospital inpatient data and returns data.frame and outputs a phenotype file with phenotypes for COVID-19 susceptibility, severity or mortality.
*comorbidity_summary*	Summarises disease history records of each individual from the hospital inpatient diagnosis data and generates a file including all comorbidities based on ICD10 code, which can be used in the comorbidity association tests.
*comorbidity_asso*	Performs association tests using logistic regression models, adjusts the tested phenotype with covariates and outputs a table comprised of odds ratios (ORs), 95% confidence intervals (CIs) of ORs, and p-values for all the comorbidity categories.
*sampleQC*	Collates genetic QC data, as provided by UKBB and outputs lists of samples for inclusion/exclusion, for use with PLINK ( Purcell *et al*., 2007) and/or SAIGE ( Zhou *et al*., 2018). Also outputs a csv file summary sample-level QC metrics.
*variantQC*	Collates genetic QC data, as provided by UKBB and outputs lists of variants for inclusion in downstream analyses, for use with PLINK and/or SAIGE.
*makeGWASFiles*	Output phenotype files, formatted to be used as input for GWAS, or other genetic analyses, with PLINK and/or SAIGE.
*log_cov*	Performs association tests using logistic regression models.

### COVID-19 test results data

COVID-19 test results data are being provided to the UKBB by Public Health England (PHE), Public Health Scotland (PHS) and SAIL Databank for English, Scottish and Welsh data respectively. The data have been updated approximately once every two weeks since 16 March 2020. Most samples tested for the COVID-19 disease-causing virus, SARS-CoV-2, are from combined nose/throat swabs. In intensive care settings, lower respiratory tract samples may also have been taken and analysed. The data consists of the encoded participant ID, date the specimen was taken, specimen type (e.g. nasal, nose and throat, sputum), the laboratory that processed the sample, whether the sample was reported as positive or negative for SARS-CoV-2, the requesting organisation description, as well as other variables. The test result data used in the analyses of this report are up to 6 April 2021.

### Death register data

The death register data includes the date of death, the primary and contributory causes of death, coded using the ICD-10 system. The death register data have been updated every one or two months. The death register data used in the analyses of this report are up to 23 March 2021.

### Hospital inpatient data

The hospital inpatient data consist of seven tables: 1) HESIN: the overall master table, providing information on admissions and discharges, the type of admission and other information related to the inpatient record as a whole. 2) HESIN_DIAG: diagnosis codes (ICD-9 or ICD-10) relating to inpatient records, including primary diagnoses and secondary diagnoses. The primary diagnosis is the main condition treated or investigated during the relevant episode. A secondary diagnosis is a clinically relevant contributory factor or issue that impacts the primary diagnosis (including chronic conditions). 3) HESIN_OPER: operations and procedures codes (OPCS-3 or OPCS-4) relating to inpatient episodes. 4) HESIN_CRITICAL: a child table of HESIN containing further information about those hospital episodes that required treatment in a critical care unit. 5) HESIN_PSYCH: a sibling table to HESIN containing fields relating to administrative aspects of psychiatric admissions. 6) HESIN_MATERNITY: a sibling table to HESIN containing fields relating specifically to maternity admissions. 7) HESIN_DELIVERY: Information regarding a child born as a result of a HESIN_MATERNITY record, where applicable. In this study, we use the HESIN, the HESIN_DIAG, the HESIN_OPER, and the HESIN_CRITICAL tables. The hospital inpatient data used in the analyses of this report are up to 5 February 2021.

### Phenotype definition

The ***makePhenotypes*** function defines multiple COVID-19 traits, related to susceptibility, severity and mortality, which may be used for association testing and GWAS ( Table 2).

**Table 2.** The COVID-19 related phenotypes output from the *makePhenotypes* function in the UKB.COVID19 R package.

Category	Trait Variable	Description
susceptibility	pos.neg	COVID-19 case vs negative test result - binary variable. *1 = evidence of COVID-19, from one or more of: a) positive test result for SARS-CoV-2 infection; b) admitted to hospital with COVID-19; c) death with COVID-19.* *0 = no evidence of COVID-19, due to consistently testing negative for SARS-CoV-2 infection.* *NA = no evidence of COVID-19, and no record of test result for SARS-CoV-2 infection.*
	pos.ppl	COVID-19 case vs the rest of the UKBB participants - binary variable. *1 = evidence of COVID-19, from one or more of: a) positive test result for SARS-CoV-2 infection; b) admitted to hospital with COVID-19; c) death with COVID-19.* *0 = any individual, not meeting the criteria for a COVID-19 case.*
severity	hospitalisation	COVID-19 cases with hospitalisation vs the rest of COVID-19 cases - binary variable. *1 = evidence of COVID-19* severity level 1 *, from one or more of: a) admitted to hospital due to COVID-19; b) received basic critical care or advanced critical care due to COVID-19; c) death due to COVID-19.* *0 = no evidence of COVID-19* severity level 1 *, even though testing positive for SARS-CoV-2 infection.*
	critical.care	COVID-19 cases with critical care vs the rest of COVID-19 cases - binary variable. *1 = evidence of COVID-19* severity level 2 *, from one or more of: a) received basic critical care or advanced critical care due to COVID-19; c) death due to COVID-19.* *0 = no evidence of COVID-19* severity level 2 *, even though testing positive for SARS-CoV-2 infection.*
	advanced.critical.care	COVID-19 cases with severity level 3 vs the rest of COVID-19 cases - binary variable. *1 = evidence of COVID-19* severity level 3 *, from one or more of: a) received advanced critical care due to COVID-19; c) death due to COVID-19.* *0 = no evidence of COVID-19* severity level 3 *, even though testing positive for SARS-CoV-2 infection.*
mortality	mortality	COVID-19 cases who have died due to COVID-19 vs the rest of COVID-19 cases - binary variable. *1 = death due to COVID-19.* *0 = any other COVID-19 cases.*

For susceptibility analysis, we generated a proxy variable, which includes all participants who have been tested for COVID-19 and define those who received at least one positive result as cases. By 6 April 2021, 77,222 individuals in the UKBB had received COVID-19 tests and 16,562 had tested positive for COVID-19 on at least one occasion. The ***pheno.type = “susceptibility”*** option summarises the COVID-19 test results data and generates a susceptibility phenotype for association tests and GWAS.

Based on the World Health Organization (WHO) ordinal scale for clinical improvement, we classify severity into four levels. These levels are defined as 1) hospitalisation: individuals admitted to hospital with their primary diagnosis recorded as COVID-19. 2) critical care level 2: individuals required basic treatment in a critical care unit, such as non-invasive ventilation and continuous positive airway pressure, and with their primary diagnosis recorded as COVID-19. 3) critical care level 3: individuals required advanced treatment in a critical care unit, such as invasive ventilation and temporary tracheostomy, and with their primary diagnosis recorded as COVID-19. 4) mortality: individuals died due to COVID-19. The critical care information was summarised from the HESIN_CRITICAL table and the HESIN_OPER table. The critical care level 2 cases are the COVID-19 patients who required at least one “Critical care level 2 days” in the HESIN_CRIRICAL table or received basic respiratory support, such as, E85.2 non-invasive ventilation NEC, in the HESIN_OPER table. The critical care level 3 cases are defined as the COVID-19 patients who required at least one “Critical care level 3 days” in the HESIN_CRIRICAL table or received advanced respiratory support, such as, E85.1 invasive ventilation, in the HESIN_OPER table. The commonly used GWAS tools, such as SAIGE and PLINK, do not support ordinal categorical phenotypes. Therefore, we converted this ordinal variable into four binary variables named “hospitalisation”, “critical care”, “advanced critical care” and “mortality” ( Table 2). However, users can get the ordinal variable by simply summing the four binary variables. We assume that participants who were tested COVID-19 positive but did not admit to hospital had no or mild symptoms and hence classified them as controls in severity phenotypes. We compare the test results data and the hospital inpatient data and correct any inconsistency between the two tables. As an example of data inconsistency, up to 5 February 2021, 130 individuals were admitted to the hospital due to COVID-19 but are not recorded in the test result data, while 33 individuals were admitted to the hospital due to COVID-19 but received basic negative COVID-19 test results. This inconsistency is resolved by retaining all 163 individuals and setting their COVID-19 test results as positive. The ***pheno.type = “severity”*** option combines COVID-19 test results data and hospital inpatient data and generates three phenotypes for each severity level.

For mortality, we include all individuals who received at least one positive test result and define those whose primary cause of death is recorded as being due to COVID-19 as cases. We also compare the test results data and the death register data and correct any inconsistencies. As an example, up to 23 March 2021, 205 individuals died from COVID-19 as reported by the death register data but are not recorded as having positive COVID-19 tests in the test result data while 39 individuals died from COVID-19 but received negative COVID-19 test results. The inconsistency is resolved by retaining all 244 individuals and setting their test results as positive. Therefore, in total 1,042 UKBB participants had died from COVID-19 by 23 March 2021. The ***pheno.type = “mortality”*** option combines the COVID-19 test results data and death register data and generates a mortality phenotype.

The ***makePhenotypes*** function returns results in data.frame format and outputs files in text format for the downstream association tests and genome-wide association tests using PLINK (RRID:SCR_001757) ( Purcell *et al*., 2007) and SAIGE (Scalable and Accurate Implementation of GEneralized mixed model) ( Zhou *et al*., 2018).

### Non-genetic risk factors

The ***risk_factor*** function generates formatted variables for several non-genetic risk factors from the linked health data provided by UKBB. These variables are all established risk factors for SARS-CoV-2 exposure, and/or COVID-19 severity ( Pijls *et al*., 2021; Wolff *et al*., 2021; Booth *et al*., 2021). The currently selected risk factors are listed in Table 3. The multi-category variables are converted into multiple dummy variables. For the blood type group factor, three dummy variables encoding the blood types A, AB, and O, are added to the data to compare with blood type B (baseline). For the ethnic background factor, Black, Asian, Mixed, and other ethnic backgrounds (BAME) are added to the data to permit comparison to white Europeans (baseline).

**Table 3.** The current selected risk factors of COVID-19 in the UKB.COVID19 R package.

Risk-factor variable	Description
sex	Participant sex. Binary variable 1 = male 0 = female
age	Age of participant (at 2020 birthday). Numeric
bmi	Body mass index. Numeric Where multiple longitudinal BMI measurements are available, the most recently recorded value is used.
ethnic	Self-reported “ethnic group”. Categorical 1 = White, 1001 = British, 1002 = Irish, 1003 = Any other white background. 2 = Mixed, 2001 = White and Black Caribbean, 2002 = White and Black African, 2003 = White and Asian, 2004 = Any other mixed background. 3 = Asian or Asian British, 3001 = Indian, 3002 = Pakistani, 3003 = Bangladeshi, 3004 = Any other Asian background. 5 = Chinese. 4 = Black or Black British, 4001 = Caribbean, 4002 = African, 4003 = Any other Black background. 6 = Other ethinic group. -1 = Do not know. -3 = Prefer not to answer.
other.ppl	Participant self-reports as “Other ethnic group”. Binary variable 1 = Yes 0 = No
black	Participant self-reports as “Black or Black British”. Binary variable 1 = Yes 0 = No
asian	Participant self-reports as “Asian or Asian British”. Binary variable 1 = Yes 0 = No
mixed	Participant self-reports as “Mixed”. Binary variable 1 = Yes 0 = No
white	Participant self-reports as “White”. Binary variable 1 = Yes 0 = No
SES	Socioeconomic status (SES) using a Townsend deprivation index (Black 1988). Numeric For the population of a given area, a Townsend deprivation score is the summation of Z scores of four variables: unemployment, non-car ownership, non-home ownership and household overcrowding. A greater Townsend index score implies a greater degree of deprivation. Z scores = (percentage – mean of all percentages)/SD of all percentages.
smoke	Pack-years of smoking. Numeric Where multiple longitudinal pack-years measurements are available, the most recently recorded value is used. Number of cigarettes per day/20 * (Age stopped smoking - Age start smoking) Note: Individuals who started and gave up smoking before 16 years of age were coded as NA. For individuals who started smoking before 16 but gave up after 16, their age start was set as 16. Individuals who reported starting and stopping smoking at the same age and reported giving up smoking for more than 6 months had pack-years set at 0.
blood group	Participant blood type. Categorical Participants' blood groups were extracted from imputed genotyped data (Field 23165), which was added in July 2020 as a result of the suggestion that blood group may affect COVID-19 outcomes. Blood groups: AA, AB, AO, BB, BO, OO.
O	Participant has O-type blood. Binary variable 1 = Yes 0 = No
AB	Participant has AB-type blood. Binary variable 1 = Yes 0 = No
B	Participant has B-type blood. Binary variable 1 = Yes 0 = No
A	Participant has A-type blood. Binary variable 1 = Yes 0 = No
inAgedCare	Evidence that the participant resides in an Aged Care facility. Binary variable. 1 = Evidence of residing in aged care, based on HES data (admitted from, or discharged to, a nursing, residential care, group home), or from the COVID-19 test data (requesting organisation). 0 = Any individual not having evidence for residing in aged care, as defined above.

Simple associations between COVID-19 phenotypes and these common risk factors may be examined using the ***log_cov*** function, which performs a logistic regression model and formats the results for quick interpretation.

### Comorbidities

The ***comorbidity_summary*** function summarises disease history records of each individual from the hospital inpatient diagnosis data. To meet different research aims the function allows restriction to a period and filtering of annotations by only primary diagnoses or all diagnoses (using the "Date.start", "Date.end" and "primary" arguments, respectively). For illustration, if we are interested in the co-occurrences of COVID-19, we can set the episode start date as 16 March 2020 (“Date.start = 16/03/2020”), when the first COVID-19 test result was recorded and choose to use all diagnoses (“primary = FALSE”). If we are interested in individuals with reported comorbidities that are at a higher risk to SARS-CoV-2, we can choose an episode start time before the COVID-19 outbreak in the UK, for example, “Date.end = 01/01/2020” and only focus on the primary diagnoses (“primary = TRUE”). Comorbidity categories are generated using the block categories in the ICD10 code, which is shown in the second column in Table 4. We include ICD10 chapters 1-14 and 17 and exclude several chapters such as pregnancy, childbirth, and consequences of external causes etc. For instance, the first category is “A00-A09”, representing intestinal infectious diseases. During a period restricted by the start and end dates, cases are defined as any participants who were diagnosed as any subclasses under the block A00‐A09 in the hospital inpatient diagnosis data. In this way, 164 binary variables are generated and each of them represents a comorbidity category. The R function generates a text file including all comorbidity categories, which can be used in the comorbidity association tests.

**Table 4.** The comorbidity categories. Comorbidity categories are generated using the block categories in the ICD10 code, as shown in the second column. We only included the blocks in chapter 1-14 and 17 and excluded several chapters such as pregnancy, childbirth and consequences of external causes etc.

Chapter	Block	Title
I	Block A00-A09	Intestinal infectious diseases
	Block A15-A19	Tuberculosis
	Block A20-A28	Certain zoonotic bacterial diseases
	Block A30-A49	Other bacterial diseases
	Block A50-A64	Infections with a predominantly sexual mode of transmission
	Block A65-A69	Other spirochaetal diseases
	Block A70-A74	Other diseases caused by chlamydiae
	Block A75-A79	Rickettsioses
	Block A80-A89	Viral infections of the central nervous system
	Block A92-A99	Arthropod-borne viral fevers and viral haemorrhagic fevers
II	Block B00-B09	Viral infections characterized by skin and mucous membrane lesions
	Block B15-B19	Viral hepatitis
	Block B20-B24	Human immunodeficiency virus [HIV] disease
	Block B25-B34	Other viral diseases
	Block B35-B49	Mycoses
	Block B50-B64	Protozoal diseases
	Block B65-B83	Helminthiases
	Block B85-B89	Pediculosis, acariasis and other infestations
	Block B90-B94	Sequelae of infectious and parasitic diseases
	Block B95-B98	Bacterial, viral and other infectious agents
	Block B99-B99	Other infectious diseases
III	Block C00-C14	Malignant neoplasms of lip, oral cavity and pharynx
	Block C15-C26	Malignant neoplasms of digestive organs
	Block C30-C39	Malignant neoplasms of respiratory and intrathoracic organs
	Block C40-C41	Malignant neoplasms of bone and articular cartilage
	Block C43-C44	Melanoma and other malignant neoplasms of skin
	Block C45-C49	Malignant neoplasms of mesothelial and soft tissue
	Block C50-C50	Malignant neoplasm of breast
	Block C51-C58	Malignant neoplasms of female genital organs
	Block C60-C63	Malignant neoplasms of male genital organs
	Block C64-C68	Malignant neoplasms of urinary tract
	Block C69-C72	Malignant neoplasms of eye, brain and other parts of central nervous system
	Block C73-C75	Malignant neoplasms of thyroid and other endocrine glands
	Block C76-C80	Malignant neoplasms of ill-defined, secondary and unspecified sites
	Block C81-C96	Malignant neoplasms, stated or presumed to be primary, of lymphoid, haematopoietic and related tissue
	Block C97-C97	Malignant neoplasms of independent (primary) multiple sites
IV	Block D00-D09	In situ neoplasms
	Block D10-D36	Benign neoplasms
	Block D37-D48	Neoplasms of uncertain or unknown behaviour
	Block D50-D53	Nutritional anaemias
	Block D55-D59	Haemolytic anaemias
	Block D60-D64	Aplastic and other anaemias
	Block D65-D69	Coagulation defects, purpura and other haemorrhagic conditions
	Block D70-D77	Other diseases of blood and blood-forming organs
	Block D80-D89	Certain disorders involving the immune mechanism
V	Block E00-E07	Disorders of thyroid gland
	Block E10-E14	Diabetes mellitus
	Block E15-E16	Other disorders of glucose regulation and pancreatic internal secretion
	Block E20-E35	Disorders of other endocrine glands
	Block E40-E46	Malnutrition
	Block E50-E64	Other nutritional deficiencies
	Block E65-E68	Obesity and other hyperalimentation
	Block E70-E90	Metabolic disorders
VI	Block F00-F09	Organic, including symptomatic, mental disorders
	Block F10-F19	Mental and behavioural disorders due to psychoactive substance use
	Block F20-F29	Schizophrenia, schizotypal and delusional disorders
	Block F30-F39	Mood [affective] disorders
	Block F40-F48	Neurotic, stress-related and somatoform disorders
	Block F50-F59	Behavioural syndromes associated with physiological disturbances and physical factors
	Block F60-F69	Disorders of adult personality and behaviour
	Block F70-F79	Mental retardation
	Block F80-F89	Disorders of psychological development
	Block F90-F98	Behavioural and emotional disorders with onset usually occurring in childhood and adolescence
	Block F99-F99	Unspecified mental disorder
VII	Block G00-G09	Inflammatory diseases of the central nervous system
	Block G10-G14	Systemic atrophies primarily affecting the central nervous system
	Block G20-G26	Extrapyramidal and movement disorders
	Block G30-G32	Other degenerative diseases of the nervous system
	Block G35-G37	Demyelinating diseases of the central nervous system
	Block G40-G47	Episodic and paroxysmal disorders
	Block G50-G59	Nerve, nerve root and plexus disorders
	Block G60-G64	Polyneuropathies and other disorders of the peripheral nervous system
	Block G70-G73	Diseases of myoneural junction and muscle
	Block G80-G83	Cerebral palsy and other paralytic syndromes
	Block G90-G99	Other disorders of the nervous system
VIII	Block H00-H06	Disorders of eyelid, lacrimal system and orbit
	Block H10-H13	Disorders of conjunctiva
	Block H15-H22	Disorders of sclera, cornea, iris and ciliary body
	Block H25-H28	Disorders of lens
	Block H30-H36	Disorders of choroid and retina
	Block H40-H42	Glaucoma
	Block H43-H45	Disorders of vitreous body and globe
	Block H46-H48	Disorders of optic nerve and visual pathways
	Block H49-H52	Disorders of ocular muscles, binocular movement, accommodation and refraction
	Block H53-H54	Visual disturbances and blindness
	Block H55-H59	Other disorders of eye and adnexa
	Block H60-H62	Diseases of external ear
	Block H65-H75	Diseases of middle ear and mastoid
	Block H80-H83	Diseases of inner ear
	Block H90-H95	Other disorders of ear
IX	Block I00-I02	Acute rheumatic fever
	Block I05-I09	Chronic rheumatic heart diseases
	Block I10-I15	Hypertensive diseases
	Block I20-I25	Ischaemic heart diseases
	Block I26-I28	Pulmonary heart disease and diseases of pulmonary circulation
	Block I30-I52	Other forms of heart disease
	Block I60-I69	Cerebrovascular diseases
	Block I70-I79	Diseases of arteries, arterioles and capillaries
	Block I80-I89	Diseases of veins, lymphatic vessels and lymph nodes, not elsewhere classified
	Block I95-I99	Other and unspecified disorders of the circulatory system
X	Block J00-J06	Acute upper respiratory infections
	Block J09-J18	Influenza and pneumonia
	Block J20-J22	Other acute lower respiratory infections
	Block J30-J39	Other diseases of upper respiratory tract
	Block J40-J47	Chronic lower respiratory diseases
	Block J60-J70	Lung diseases due to external agents
	Block J80-J84	Other respiratory diseases principally affecting the interstitium
	Block J85-J86	Suppurative and necrotic conditions of lower respiratory tract
	Block J90-J94	Other diseases of pleura
	Block J95-J99	Other diseases of the respiratory system
XI	Block K00-K14	Diseases of oral cavity, salivary glands and jaws
	Block K20-K31	Diseases of oesophagus, stomach and duodenum
	Block K35-K38	Diseases of appendix
	Block K40-K46	Hernia
	Block K50-K52	Noninfective enteritis and colitis
	Block K55-K64	Other diseases of intestines
	Block K65-K67	Diseases of peritoneum
	Block K70-K77	Diseases of liver
	Block K80-K87	Disorders of gallbladder, biliary tract and pancreas
	Block K90-K93	Other diseases of the digestive system
XII	Block L00-L08	Infections of the skin and subcutaneous tissue
	Block L10-L14	Bullous disorders
	Block L20-L30	Dermatitis and eczema
	Block L40-L45	Papulosquamous disorders
	Block L50-L54	Urticaria and erythema
	Block L55-L59	Radiation-related disorders of the skin and subcutaneous tissue
	Block L60-L75	Disorders of skin appendages
	Block L80-L99	Other disorders of the skin and subcutaneous tissue
XIII	Block M00-M03	Infectious arthropathies
	Block M05-M14	Inflammatory polyarthropathies
	Block M15-M19	Arthrosis
	Block M20-M25	Other joint disorders
	Block M40-M43	Deforming dorsopathies
	Block M45-M49	Spondylopathies
	Block M50-M54	Other dorsopathies
	Block M60-M63	Disorders of muscles
	Block M65-M68	Disorders of synovium and tendon
	Block M70-M79	Other soft tissue disorders
	Block M80-M85	Disorders of bone density and structure
	Block M86-M90	Other osteopathies
	Block M91-M94	Chondropathies
	Block M95-M99	Other disorders of the musculoskeletal system and connective tissue
XIV	Block N00-N08	Glomerular diseases
	Block N10-N16	Renal tubulo-interstitial diseases
	Block N17-N19	Renal failure
	Block N20-N23	Urolithiasis
	Block N25-N29	Other disorders of kidney and ureter
	Block N30-N39	Other diseases of urinary system
	Block N40-N51	Diseases of male genital organs
	Block N60-N64	Disorders of breast
	Block N70-N77	Inflammatory diseases of female pelvic organs
	Block N80-N98	Noninflammatory disorders of female genital tract
	Block N99-N99	Other disorders of the genitourinary system
XVII	Block Q00-Q07	Congenital malformations of the nervous system
	Block Q10-Q18	Congenital malformations of eye, ear, face and neck
	Block Q20-Q28	Congenital malformations of the circulatory system
	Block Q30-Q34	Congenital malformations of the respiratory system
	Block Q35-Q37	Cleft lip and cleft palate
	Block Q38-Q45	Other congenital malformations of the digestive system
	Block Q50-Q56	Congenital malformations of genital organs
	Block Q60-Q64	Congenital malformations of the urinary system
	Block Q65-Q79	Congenital malformations and deformations of the musculoskeletal system
	Block Q80-Q89	Other congenital malformations
	Block Q90-Q99	Chromosomal abnormalities, not elsewhere classified

The ***comorbidity_asso*** function performs association tests between each comorbidity category and the selected phenotype using logistic regression models and adjusts the tested phenotype with covariates, which can be set using the argument “cov.name”. By default, the covariates include sex, age, and BMI. Different ethnic backgrounds can be chosen for the test by setting the argument “population”. By default, all populations are included. It outputs a table comprised of odds ratios (ORs), confidence intervals (CIs) of ORs, and p-values for all the comorbidity categories.

### Preparation of files for genetic analyses

The UKB.COVID19 package provides several functions, to facilitate GWAS, or other genetic analyses using the UKBB data. We provide two functions ***sampleQC*** and ***variantQC***, to allow easy cleaning of the genetic data, using quality control (QC) metrics, supplied by UKBB ( Bycroft *et al*., 2018). A third function, ***makeGWASFiles***, outputs phenotype files, which may be used as input for the GWAS software packages PLINK ( Purcell *et al*., 2007) and SAIGE ( Zhou *et al*., 2018).

The ***sampleQC*** function outputs a csv file summarising sample-level QC metrics, as well as producing lists of IDs for inclusion and/or exclusion in downstream analyses. The function identifies individuals to be excluded from genetic analyses based on: 1) being excluded by UKBB, before imputation due to high heterozygosity or missingness (>5%), 2) sex mismatches between genetically predicted and recorded sex, 3) an apparent excess number of relatives in the UKBB cohort (≥ 10 relatives), 4) putative sex chromosome aneuploidy, 5) withdrawn consent. The user has the option of further restricting to individuals of “White British” ancestry (determined using genetic principal components), by using the ancestry argument. Finally, the user can specify whether they require inclusion/exclusion sample lists to be formatted for PLINK or SAIGE.

The ***variantQC*** function identifies variants to be included in downstream analyses, based on minor allele frequency (MAF) and imputation quality (INFO score), with thresholds specified by the user (defaults to MAF ≥0.001 and INFO ≥0.5). The function outputs list of variants passing these thresholds are in two formats, given the two types of SNP IDs available in the UKBB imputed genetic data release: 1) *snpIncludeSNPIDs_minMaf0.001_minInfo0.5.txt* contains the unique SNP identifiers; 2) *snpIncludeRSIDs_minMaf0.001_minInfo0.5.txt contains the* rsid or the reference panel marker ID (note these IDs are not guaranteed to be unique). The function also outputs a file containing IDs of the subset of SNPs, used by UKBB for calculating ancestry principal components ( Bycroft *et al*., 2018). This subset of SNPs is suitable for analyses where a pruned set of independent SNPs are preferred, for example for calculation of a genetic relatedness matrix (GRM).

The ***makeGWASFiles*** function generates a phenotype file, suitable to be used in association analyses by either SAIGE or PLINK ( Purcell *et al*., 2007) (File format specified by user). The function utilises the phenotypes data frame generated by the ***makePhenotypes*** function, with the user able to specify specific phenotypes. The output phenotype file also contains the first 20 ancestry principal components, and genotyping array, as these are likely to be required as covariates in any genetic analyses. The user can also specify additional covariates (e.g. those generated by the ***risk_factor*** function), to be outputted to the phenotype file. Finally, the user can choose to output phenotypes, only for the individuals passing all QC (using the output file from ***sampleQC*** function), or for all individuals.

### GWAS

We performed QC for the genotype data from UKBB using the ***sampleQC*** function, with the ancestry = “WhiteBritish” option, and the ***variantQC*** function, with thresholds MAF = 0.01 and INFO = 0.8. Phenotype files for SAIGE were generated using the ***makeGWASFiles*** function, containing all variables generated by the ***risk_factor*** function.

Using the output files from the ***sampleQC*** and ***variantQC*** functions, we filtered the directly genotyped data using PLINK ( Purcell *et al*., 2007), and the imputed data using QCTool version 2. We then performed GWAS of all COVID-19 phenotypes using SAIGE ( Zhou *et al*., 2018). Firstly, the null model was fitted for each phenotype with 20 ancestry procedure codes (PCs), genotypic array, and associated non-genetic risk factors as covariates, and we used the pruned subset SNPs to construct the GRM. Subsequently, genome-wide association testing was undertaken, using the filtered imputed data.

### Statistical analysis

To assess the associations between non-genetic risk factors and COVID-19 phenotypes (including susceptibility, severity, and mortality), we employed multivariable logistic regression models using the ‘glm’ function from the R package stats. Each model adjusted for covariates such as age, sex, and BMI. The tested risk factors included socioeconomic status (SES), smoking status, blood type, ethnic background, and residence in aged care facilities. The logistic regression model for each risk factor was specified as follows: logit (COVID-19 phenotype) ~ risk factor + age + sex + BMI. Comorbidity associations were analyzed using similar multivariable logistic regression models, with COVID-19 phenotypes modeled as: logit (COVID-19 phenotype) ~ comorbidity category + age + sex + BMI + SES + smoking status + aged care status. Odds ratios (ORs) with 95% confidence intervals (CIs) were reported, and p-values were calculated to determine the significance of the associations.

To identify genetic variants associated with COVID-19 phenotypes, we performed GWASs using the SAIGE software. Principal component analysis (PCA) was performed to account for population stratification, and the first 20 principal components (PCs) were included as covariates in the analysis. Additionally, we adjusted for age, sex, BMI, SES, smoking status, residence in aged care facilities and genotypic array in the regression models. The association between each SNP and the phenotypes was tested using a logistic regression model, as follows: logit (COVID-19 phenotype) ~ SNP + age + sex + BMI + SES + smoking status + aged care status + genotypic array + PC1-20. To account for multiple testing, the Bonferroni correction was applied. Loci reaching the genome-wide significance threshold (p < 5×10 ^−8^) were considered significant. Manhattan plots and quantile-quantile (QQ) plots were generated to visualize the results using R package ggplot2. All analyses were carried out using R (version 4.0.5).

## Results

We applied the R package UKB.COVID19 to the data released in April 2021. The last records in the COVID-19 test results data, the death register data and the hospital inpatient data were recorded on 6 April 2021, 23 March 2021, and 5 February 2021, respectively. By default, the dates for susceptibility, severity and mortality studies were chosen as 6 April 2021, 5 February 2021, and 23 March 2021, accordingly.

### COVID-19 susceptibility

By 6 April 2021, 77,222 UKBB participants had tested for COVID-19. Among these individuals, 16,562 received at least one positive test result and 60,660 received all negative results. First, we tested the associations between a positive test result (as a proxy for COVID-19 susceptibility), and age, sex, and BMI using multivariable logistic regression. The results ( Table 5) show increased odds of a positive result in individuals of male sex (OR = 1.08, 95% CI = [1.04,1.11], p-value = 0.00007), with higher BMI (OR = 1.026, 95% CI = [1.0229,1.03], p-value <10 ^−5^) and with younger ages (OR = 0.939, 95% CI = [0.937,0.941], p-value <10 ^−5^). A possible reason for this result is that the older participants are less active and thus had less chance of being exposed to SARS-CoV-2.

**Table 5.** COVID-19 susceptibility and non-genetic risk factor association test results for all populations and white British. Cases are defined as participants who received at least one COVID-19 positive test result. Controls are those who received only negative results. We tested sex, age and body mass index (BMI) in a multivariable model first and then tested each other factor individually by adjusting sex, age and BMI. SES stands for socioeconomic status. Odds ratio (OR) and p-values (P) are provided.

Samples	Case/control	Statistic	Sex	Age	BMI	Blood type			Ethnic background				inAgedCare	SES	Smoke
						A	AB	O	Black	Asian	Mixed	Other			
All populations	16,562/60,660	OR	1.08	0.94	1.03	0.99	1.09	0.91	1.38	1.88	1.02	1.33	2.13	1.04	1.003
		P	0.00007	≈0	≈0	0.7	0.1	0.005	≈0	≈0	0.9	0.0004	≈0	≈0	≈0
White British	14,767/57,068	OR	1.07	0.94	1.03	1.05	1.10	0.96					2.36	1.04	1.004
		P	0.0008	≈0	≈0	0.2	0.1	0.2					≈0	≈0	≈0

Baseline: sex - female; blood type - B; ethnic background - white.

*≈0 means <10 ^−5^.

Second, we tested each potential risk factor individually with adjustment of age, sex, and BMI. Several publications have already reported that blood type groups are associated with COVID-19 susceptibility ( Zhao *et al*., 2020; Zietz, Zucker, and Tatonetti 2020), including genetic associations with the ABO blood group locus at 9q34.2 (The Severe Covid-19 GWAS Group “Genomewide Association Study of Severe Covid-19 with Respiratory Failure” 2020). People with blood type A have been consistently reported as being at a higher risk to SARS-CoV-2 and people with blood type O at lower risk ( Zhao *et al*., 2020). Consistent with these results we find that compared with type B, individuals with blood type O are less susceptible to SARS-CoV-2 (OR =0.91, 95% CI = [0.86,0.97], p-value = 0.005) but we were unable to replicate the type A findings (p-value = 0.7).

Compared with white individuals, those who self-identified as Black (OR =1.38, 95% CI = [1.24,1.55], p-value <10 ^−5^), Asian (OR =1.88, 95% CI = [1.71,2.07], p-value <10 ^−5^) and other ethnic backgrounds (OR =1.33, 95% CI = [1.14,1.55], p-value =0.0004) have higher odds of testing positive for COVID-19. Individuals with a lower socioeconomic status (SES) are also at a higher risk of COVID-19 (OR = 1.041, 95% CI = [1.036,1.047], p-value <10 ^−5^). Smoking also contributes to COVID-19 susceptibility (OR =1.003, 95% CI = [1.002,1.004], p-value <10 ^−5^). People who are staying at an aged care home are at a significantly higher risk of COVID-19 (OR = 2.13, 95% CI = [1.87,2.43], p-value <10 ^−5^), which is in line with the aged care home outbreaks in the UK.

**Figure 1.**
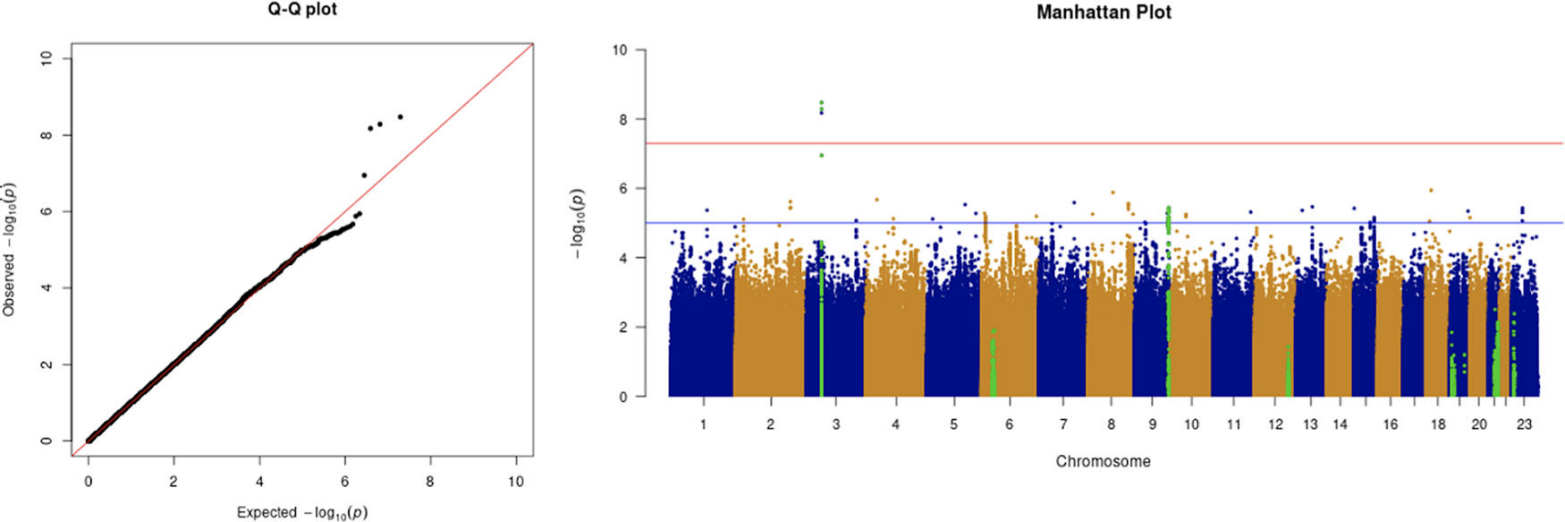
The Q-Q plot and Manhattan plot of COVID-19 susceptibility GWAS. Sample size is 61,823. In the Manhattan plot, each point denotes a SNP located on a particular chromosome ( *x*-axis). The significance level is presented in the *y*-axis. The red line indicates the threshold for genome-wide significance 5 × 10 ^−8^ while the blue line indicates the threshold for suggestive genome-wide significance 1 × 10 ^−5^. The light green dots are the genes of interest, which have been reported in other publications (Pairo-Castineira *et al*., 2021; “Genomewide Association Study of Severe Covid-19 with Respiratory Failure”, 2020), including *SLC6A20, LZTFL1, CCR9, FYCO1, CXCR6, XCR1, HLA-G, CCHCR1, NOTCH4, ABO, OAS1, OAS2, OAS3, APOE, DPP9, TYK2, IFNAR2, TMPRSS2, ACE2,* and *TLR7.* The susceptibility phenotype is adjusted by age, sex, body mass index, socioeconomic status, smoking, if in an aged care home, array, and PC1–20. The genome-wide significant COVID-19 susceptibility locus identified is 3p21.31. The most statistically significant SNP is rs2771616 within the glycine transporter gene *SLC6A20* (3p21.31, p-value =3.36 × 10 ^−9^), followed by SNPs rs73062389 (3p21.31; *SLC6A20*; p-value = 5.16 × 10 ^−9^) and rs73062394 (3p21.31; *SLC6A20*; p-value = 6.68 × 10 ^−9^) in strong linkage disequilibrium (LD) (r2 = 1 and r2 = 1).

We only apply GWAS to the white British participants in the UKBB. Therefore, we performed non-genetic risk factor association tests again for self-reported “white” participants only. It shows that age, sex, BMI, SES, smoking, and if in an aged care home are associated with COVID-19 susceptibility in white British. Incorporation of the two array effects and the first 20 PCs, these risk factors are used to adjust susceptibility in the GWAS. The genome-wide significant COVID-19 susceptibility locus identified in our GWAS is 3p21.31 ( Figure 1 and Table 6). The most statistically significant SNP is rs2771616 within the glycine transporter gene *SLC6A20* (3p21.31, p-value = 3.36 × 10 ^−9^), followed by SNPs rs73062389 (3p21.31; *SLC6A20*; p-value =5.16 × 10 ^−9^) and rs73062394 (3p21.31; *SLC6A20*; p-value = 6.68 × 10 ^−9^) in strong linkage disequilibrium (LD) (r2 = 1 and r2 = 1) ( Table 7). *SLC6A20* encodes an amino acid transporter that interacts with ACE2, the main receptor that SARS-CoV-2 uses to gain entry into host cells ( Elhabyan *et al*., 2020; Hoffmann *et al*., 2020). This locus has also been previously identified by other studies (The Severe Covid-19 GWAS Group “Genomewide Association Study of Severe Covid-19 with Respiratory Failure”, 2020), several meta-analyses of which have also made use of the UKBB COVID-19 data (Host Genetics Initiative, 2021). All genome wide significant GWAS hits with gene annotations are available in Table 7.

**Table 6.** The most genome-wide significant hits of COVID-19 susceptibility, hospitalisation and critical care genome-wide association studies.

Phenotype	RsID	Chromosome	Position	Effect/non-effect allele	Cytoband	P-value	Gene
Susceptibility	rs2271616	3	45838013	G/T	p21.31	3.36E-09	*SLC6A20*
Hospitalisation	rs35044562	3	45909024	A/G	p21.31	1.55E-10	*LZTFL1*
Critical care	rs35044562	3	45909024	A/G	p21.31	2.23E-09	*LZTFL1*

**Table 7.** The genome-wide significant hits of COVID-19 susceptibility, hospitalisation and critical care genome-wide association studies.

Phenotype	RsID	Chromosome	Position	Effect/non-effect allele	Cytoband	P-value	Nearest gene
Susceptibility	rs2271616	3	45838013	G/T	p21.31	3.36E-09	*SLC6A20*
	rs73062389	3	45835417	G/A	p21.31	5.16E-09	*SLC6A20*
	rs73062394	3	45839176	A/T	p21.31	6.68E-09	*SLC6A20*
Hospitalisation	rs35896106	3	45841938	C/T	p21.31	1.15E-08	*SLC6A20*
	rs13071258	3	45843242	G/A	p21.31	2.68E-09	*SLC6A20*
	rs17763537	3	45843315	C/T	p21.31	8.91E-09	*SLC6A20*
	rs17763569	3	45843439	G/T	p21.31	8.91E-09	*SLC6A20*
	rs34668658	3	45844198	A/C	p21.31	3.53E-09	*SLC6A20*
	rs17763742	3	45846769	A/G	p21.31	4.46E-09	*SLC6A20*
	rs17712877	3	45848760	G/C	p21.31	9.41E-09	*SLC6A20*
	rs72893671	3	45850783	T/A	p21.31	5.87E-09	*SLC6A20*
	rs17713054	3	45859651	G/A	p21.31	5.46E-10	*LZTFL1*
	rs13078854	3	45861932	G/A	p21.31	5.43E-10	*LZTFL1*
	rs71325088	3	45862952	T/C	p21.31	4.61E-10	*LZTFL1*
	rs10490770	3	45864732	T/C	p21.31	5.81E-10	*LZTFL1*
	rs35624553	3	45867440	A/G	p21.31	5.67E-10	*LZTFL1*
	3:45871139_GA_G	3	45871139	GA/G	p21.31	3.24E-09	*LZTFL1*
	rs67959919	3	45871908	G/A	p21.31	5.60E-10	*LZTFL1*
	rs11385942	3	45876459	G/GA	p21.31	1.02E-09	*LZTFL1*
	rs35508621	3	45880481	T/C	p21.31	5.24E-10	*LZTFL1*
	rs34288077	3	45888690	A/G	p21.31	6.34E-10	*LZTFL1*
	rs35081325	3	45889921	A/T	p21.31	6.34E-10	*LZTFL1*
	rs35731912	3	45889949	C/T	p21.31	6.26E-10	*LZTFL1*
	rs34326463	3	45899651	A/G	p21.31	6.26E-10	*LZTFL1*
	rs76374459	3	45900634	G/C	p21.31	6.09E-09	*LZTFL1*
	rs73064425	3	45901089	C/T	p21.31	5.41E-10	*LZTFL1*
	rs13081482	3	45908116	A/T	p21.31	5.43E-10	*LZTFL1*
	rs35652899	3	45908514	C/G	p21.31	2.01E-10	*LZTFL1*
	rs35044562	3	45909024	A/G	p21.31	1.55E-10	*LZTFL1*
	rs73064431	3	45909528	C/T	p21.31	3.55E-09	*LZTFL1*
	rs13092887	3	45909644	C/A	p21.31	2.64E-09	*LZTFL1*
Critical care	rs17713054	3	45859651	G/A	p21.31	3.76E-09	*LZTFL1*
	rs13078854	3	45861932	G/A	p21.31	3.76E-09	*LZTFL1*
	rs71325088	3	45862952	T/C	p21.31	2.61E-09	*LZTFL1*
	rs10490770	3	45864732	T/C	p21.31	3.89E-09	*LZTFL1*
	rs35624553	3	45867440	A/G	p21.31	3.88E-09	*LZTFL1*
	3:45871139_GA_G	3	45871139	GA/G	p21.31	4.14E-08	*LZTFL1*
	rs67959919	3	45871908	G/A	p21.31	3.96E-09	*LZTFL1*
	rs11385942	3	45876459	G/GA	p21.31	6.89E-09	*LZTFL1*
	rs35508621	3	45880481	T/C	p21.31	3.27E-09	*LZTFL1*
	rs34288077	3	45888690	A/G	p21.31	4.25E-09	*LZTFL1*
	rs35081325	3	45889921	A/T	p21.31	4.24E-09	*LZTFL1*
	rs35731912	3	45889949	C/T	p21.31	4.01E-09	*LZTFL1*
	rs34326463	3	45899651	A/G	p21.31	4.17E-09	*LZTFL1*
	rs76374459	3	45900634	G/C	p21.31	5.34E-09	*LZTFL1*
	rs73064425	3	45901089	C/T	p21.31	3.83E-09	*LZTFL1*
	rs13081482	3	45908116	A/T	p21.31	4.38E-09	*LZTFL1*
	rs35652899	3	45908514	C/G	p21.31	3.18E-09	*LZTFL1*
	rs35044562	3	45909024	A/G	p21.31	2.23E-09	*LZTFL1*
	rs73064431	3	45909528	C/T	p21.31	3.78E-08	*LZTFL1*
	rs13092887	3	45909644	C/A	p21.31	3.47E-08	*LZTFL1*

### COVID-19 severity

By 5 February 2021, 15,666 UKBB participants received positive COVID-19 test results. 2,104 individuals had been admitted to the hospital due to COVID-19, 1,129 of these individuals received critical care treatments and 1,010 received advanced critical care treatments. The risk factor association test results are presented in Tables 8 and 9 for all populations and self-reported white individuals, respectively. Compared to white individuals, Black, Asian, and other minority ethnic groups are at a higher risk of severe COVID-19. Age, sex, BMI, SES, and smoking are also positively associated with COVID-19 severity.

**Table 8.** COVID-19 severity and non-genetic risk factor association test results for all populations. Cases of hospitalisation include participants who were admitted to hospital and whose primary diagnosis was COVID-19, received critical care treatments, or died from COVID-19. Controls are the rest of the participants who received positive test results. Cases of critical care phenotype include those who received critical care treatments due to COVID-19 or died from COVID-19. Cases of advanced critical care are defined as participants who received advanced critical care treatments or died from COVID-19. We tested sex, age and body mass index (BMI) in a multivariable model first and then tested each other factor individually by adjusting sex, age and BMI. SES stands for socioeconomic status. Odds ratio (OR) and p-values (P) are provided.

Severity	Case/control	Statistic	Sex	Age	BMI	Blood type			Ethnic background				inAgedCare	SES	Smoke
						A	AB	O	Black	Asian	Mixed	Other			
Hospitalisation	2,104/13,562	OR	1.75	1.12	1.07	0.87	0.82	0.94	2.00	1.57	1.07	1.49	2.08	1.08	1.01
		P	≈0	≈0	≈0	0.2	0.2	0.5	≈0	0.0003	0.8	0.06	0	≈0	≈0
Critical care	1,129/14,537	OR	1.93	1.14	1.07	0.96	1.06	1.11	2.14	1.64	0.56	1.39	2.46	1.07	1.009
		P	≈0	≈0	≈0	0.8	0.8	0.4	0.00001	0.003	0.3	0.3	≈0	≈0	≈0
Advanced critical care	1,010/14,656	OR	1.82	1.15	1.07	0.99	1.10	1.12	2.24	1.69	0.67	1.28	2.60	1.06	1.009
		P	≈0	≈0	≈0	0.9	0.6	0.4	0.00001	0.003	0.5	0.4	≈0	≈0	≈0

Baseline: sex - female; blood type - B; ethnic background - white.

*≈0 means <10 ^−5^.

**Table 9.** COVID-19 severity and non-genetic risk factor association test results for white British. Cases of hospitalisation include participants who were admitted to hospital and whose primary diagnosis was COVID-19, received critical care treatments, or died from COVID-19. Controls are the rest of the participants who received positive test results. Cases of critical care phenotype include those who received critical care treatments due to COVID-19 or died from COVID-19. Cases of advanced critical care are defined as participants who received advanced critical care treatments or died from COVID-19. We tested sex, age and body mass index (BMI) in a multivariable model first and then tested each other factor individually by adjusting sex, age and BMI. SES stands for socioeconomic status. Odds ratio (OR) and p-values (P) are provided.

Severity	Case/control	Statistic	Sex	Age	BMI	Blood type			inAgedCare	SES	Smoke
						A	AB	O			
Hospitalisation	1,865/12,093	OR	1.75	1.12	1.07	0.94	0.89	1.02	2.05	1.07	1.01
		P	≈0	≈0	≈0	0.6	0.5	0.8	≈0	≈0	≈0
Critical care	1,006/12,952	OR	2.00	1.14	1.07	1.41	1.21	1.28	2.54	1.06	1.01
		P	≈0	≈0	≈0	0.3	0.4	0.08	≈0	≈0	≈0
Advanced critical care	902/13,056	OR	1.90	1.16	1.07	1.19	1.29	1.34	2.68	1.05	1.01
		P	≈0	≈0	≈0	0.2	0.3	0.05	≈0	0.00001	≈0

Baseline: sex – female; blood type – B.

*≈0 means <10 ^−5^.

**Figure 2.**
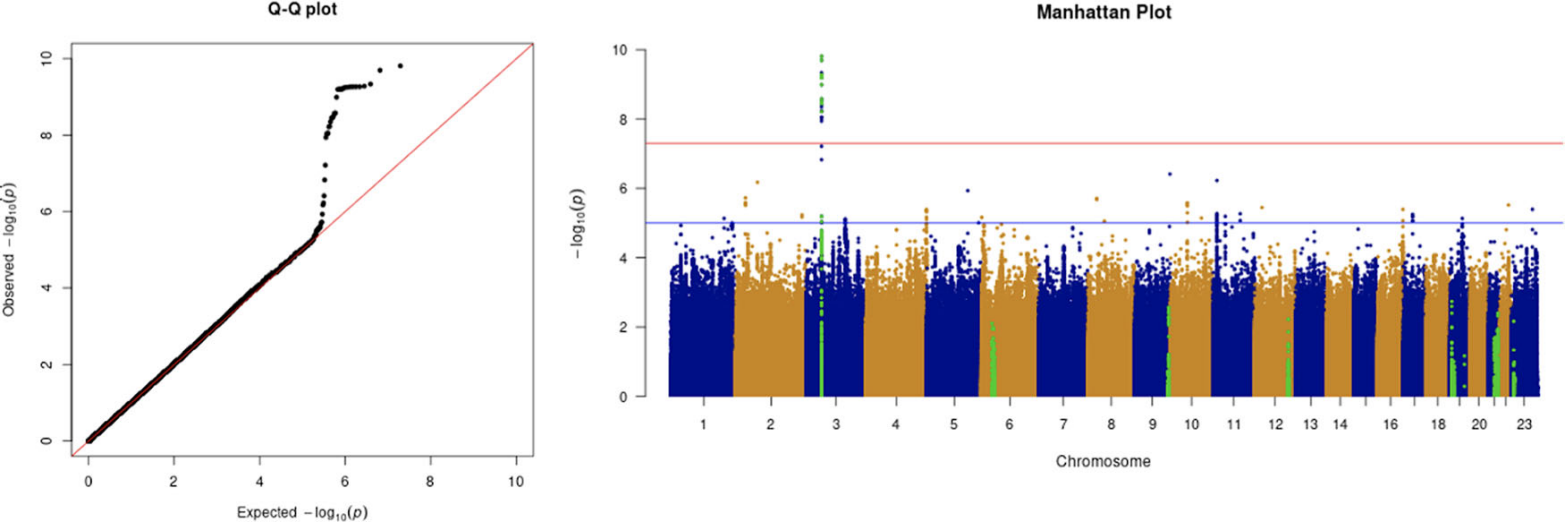
The Q-Q plot and Manhattan plot of COVID-19 hospitalisation GWAS. Sample size is 11,974. In the Manhattan plot, each point denotes a SNP located on a particular chromosome ( *x*-axis). The significance level is presented in the *y*-axis. The red line indicates the threshold for genome-wide significance 5 × 10 ^−8^ while the blue line indicates the threshold for suggestive genome-wide significance 1 × 10 ^−5^. The light green dots are the genes of interest, including *SLC6A20, LZTFL1, CCR9, FYCO1, CXCR6, XCR1, HLA-G, CCHCR1, NOTCH4, ABO, OAS1, OAS2, OAS3, APOE, DPP9, TYK2, IFNAR2, TMPRSS2, ACE2,* and *TLR7.* The hospitalisation phenotype is adjusted by age, sex, body mass index, socioeconomic status, smoking, if in an aged care home, array, and PC1–20. The result shows that the locus at 3p21.31 is genome-wide significantly associated with COVID-19 hospitalisation. The most significant SNP for both COVID-19 hospitalisation GWAS is located in the gene *LZTFL1* (rs35044562 in locus 3p21.31; p-value = 1.55 × 10 ^−10^).

**Figure 3.**
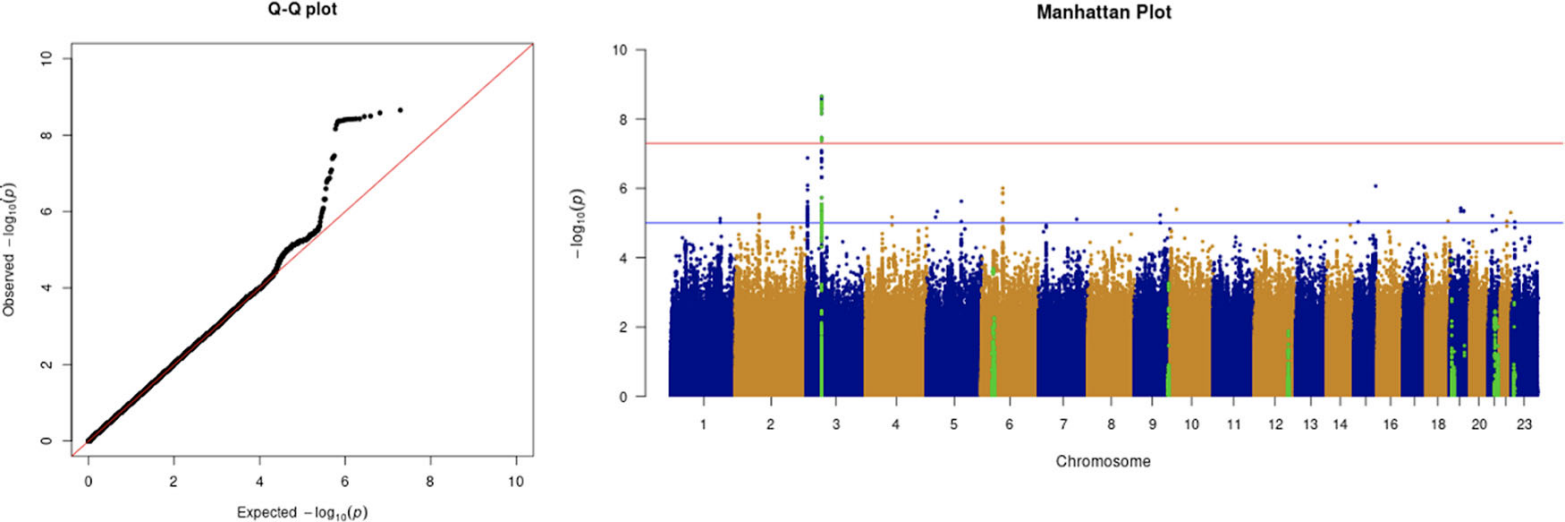
The Q-Q plot and Manhattan plot of COVID-19 critical care GWAS. Sample size is 11,974. In the Manhattan plot, each point denotes a SNP located on a particular chromosome ( *x*-axis). The significance level is presented in the *y*-axis. The red line indicates the threshold for genome-wide significance 5 × 10 ^−8^ while the blue line indicates the threshold for suggestive genome-wide significance 1 × 10 ^−5^. The light green dots are the genes of interest, including *SLC6A20, LZTFL1, CCR9, FYCO1, CXCR6, XCR1, HLA-G, CCHCR1, NOTCH4, ABO, OAS1, OAS2, OAS3, APOE, DPP9, TYK2, IFNAR2, TMPRSS2, ACE2,* and *TLR7.* The critical care phenotype is adjusted by age, sex, body mass index, socioeconomic status, smoking, if in an aged care home, array, and PC1–20. The result shows that the locus at 3p21.31 is genome-wide significantly associated with COVID-19 critical care. The most significant SNP for both COVID-19 critical care GWAS is located in the gene *LZTFL1* (rs35044562 in locus 3p21.31; p-value = 2.23 × 10 ^−9^).

**Figure 4.**
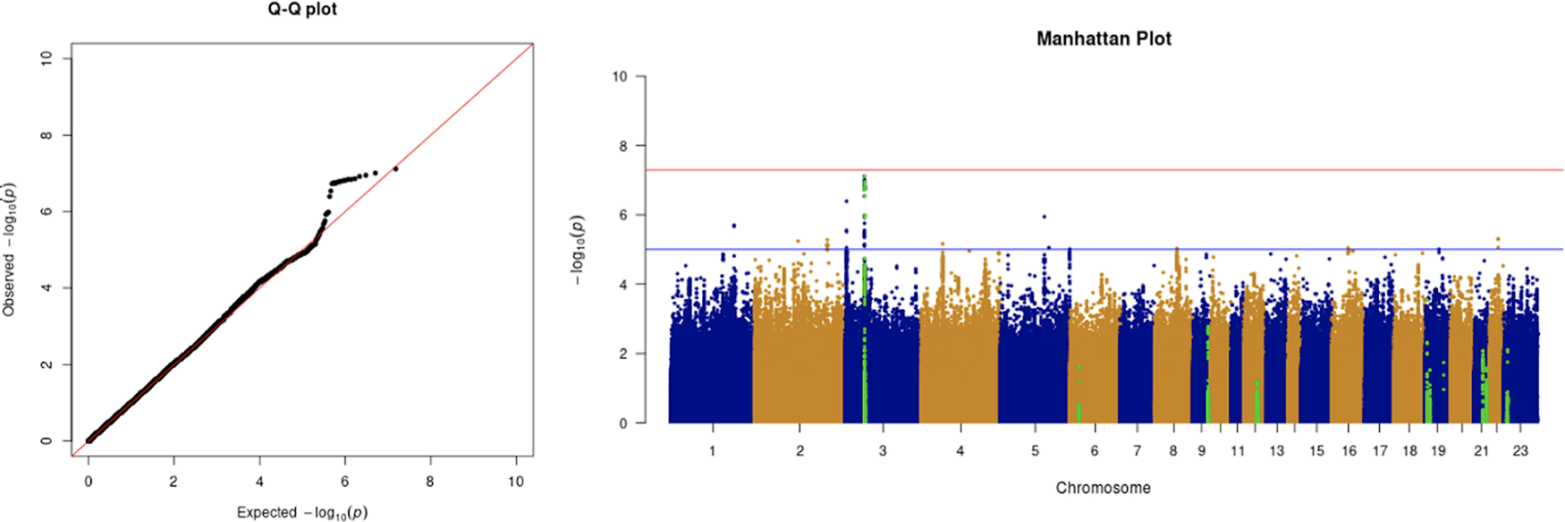
The Q-Q plot and Manhattan plot of COVID-19 advanced critical care GWAS. Sample size is 11,974. In the Manhattan plot, each point denotes a SNP located on a particular chromosome ( *x*-axis). The significance level is presented in the *y*-axis. The red line indicates the threshold for genome-wide significance 5 × 10 ^−8^ while the blue line indicates the threshold for suggestive genome-wide significance 1 × 10 ^−5^. The light green dots are the genes of interest, including *SLC6A20, LZTFL1, CCR9, FYCO1, CXCR6, XCR1, HLA-G, CCHCR1, NOTCH4, ABO, OAS1, OAS2, OAS3, APOE, DPP9, TYK2, IFNAR2, TMPRSS2, ACE2,* and *TLR7.* The advanced critical care phenotype is adjusted by age, sex, body mass index, socioeconomic status, smoking, if in an aged care home, array, and PC1–20. No genome-wide significant signals were found.

The results from the GWAS are shown in the quantile-quantile (Q-Q) plots and Manhattan plots in Figures 2–4. The tested phenotypes are adjusted by age, sex, BMI, SES, smoking, if in an aged care home, array, and PC1–20. The results show that the locus at 3p21.31 is genome-wide significantly associated with COVID-19 hospitalisation and critical care ( Tables 6 and 7). Specifically, the most significant SNP for both COVID-19 hospitalisation and critical care GWASs is located in the gene *LZTFL1* (rs35044562 in locus 3p21.31; p-value = 1.55 × 10 ^−10^ and p-value = 2.23 × 10 ^−9^, respectively). According to the Genotype-Tissue Expression ( GTEx) project, *LZTFL1* is widely expressed throughout the body and encodes a protein involved in protein trafficking to primary cilia, which are microtubule-based subcellular organelles acting as antennas for extracellular signals. In T lymphocytes, *LZTFL1* participates in the immunologic synapse with antigen-presenting cells, such as dendritic cells (these cells prime T-lymphocyte responses) ( Kaser 2020; Seo *et al*., 2011; Jiang *et al*., 2016).

### COVID-19 mortality

**Figure 5.**
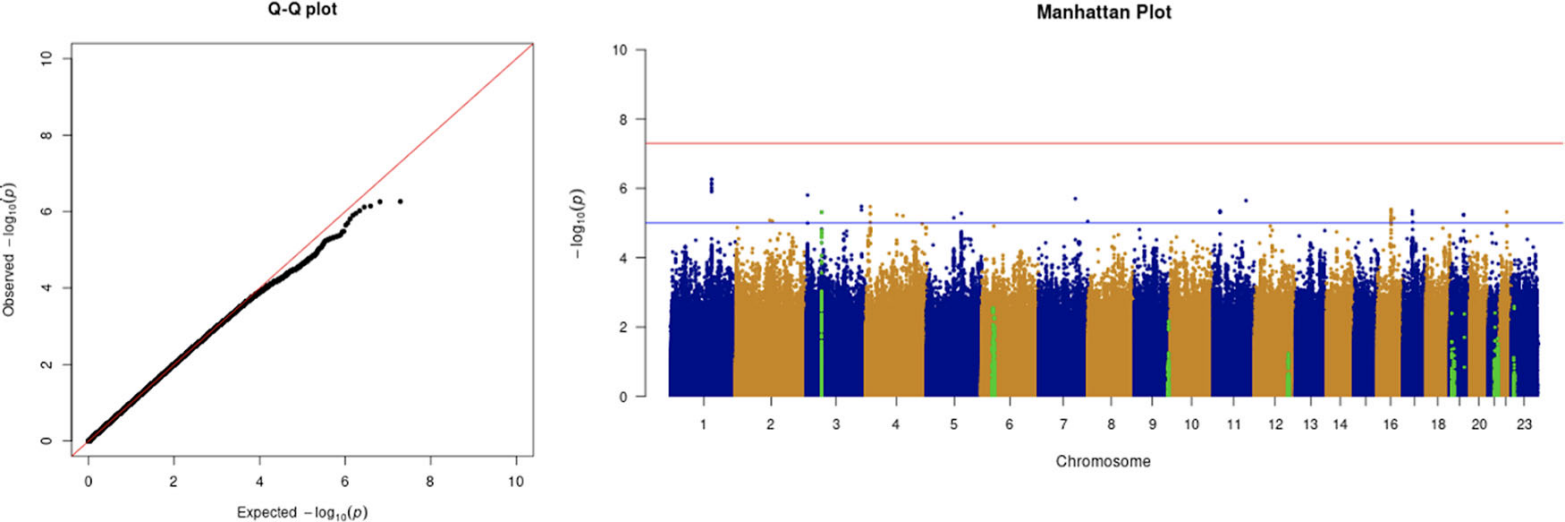
The Q-Q plot and Manhattan plot of COVID-19 mortality GWAS. Sample size is 12,790. In the Manhattan plot, each point denotes a SNP located on a particular chromosome ( *x*-axis). The significance level is presented in the *y*-axis. The red line indicates the threshold for genome-wide significance 5 × 10 ^−8^ while the blue line indicates the threshold for suggestive genome-wide significance 1 × 10 ^−5^. The light green dots are the genes of interest, including *SLC6A20, LZTFL1, CCR9, FYCO1, CXCR6, XCR1, HLA-G, CCHCR1, NOTCH4, ABO, OAS1, OAS2, OAS3, APOE, DPP9, TYK2, IFNAR2, TMPRSS2, ACE2,* and *TLR7.* The mortality phenotype is adjusted by age, sex, body mass index, socioeconomic status, smoking, if in an aged care home, array, and PC1–20. No genome-wide significant signals were found.

By 23 March 2021, 16,465 UKBB participants received positive COVID-19 test results. Among these, 1,042 individuals died from COVID-19. We performed the same association tests for COVID-19 mortality as for susceptibility and severity. The results ( Table 10) show that males have a much higher chance of dying from COVID-19 than females (OR = 1.89, 95% CI = [1.63,2.20], p-value <10 ^−5^), consistent with previously published results from independent cohorts ( Peckham *et al*., 2020). The black ethnic group is at a much higher mortality risk from SARS-CoV-2 compared to white individuals (OR = 2.04, 95% CI = [1.38,2.94], p-value = 0.0002). Age, BMI, SES, and smoking are positively associated with COVID-19 mortality. People living in aged care homes are at a much higher risk of dying from COVID-19. For self-reported white individuals, age, sex, BMI, SES, smoking, and being in an aged care home are positively associated with COVID-19 mortality. Therefore, all these covariates were used to adjust the mortality phenotype for GWAS. However, no genome-wide significant signal was detected for this GWAS ( Figure 5).

**Table 10.** COVID-19 mortality and non-genetic risk factor association test results for all populations and white British. Cases of mortality include participants whose primary death cause is COVID-19. Controls are the rest of the participants who received positive test results. We tested sex, age and body mass index (BMI) in a multivariable model first and then tested each other factor individually by adjusting sex, age and BMI. SES stands for socioeconomic status. Odds ratio (OR) and p-values (P) are provided.

Samples	Case/control	Statistic	Sex	Age	BMI	Blood type			Ethnic background				inAgedCare	SES	Smoke
						A	AB	O	Black	Asian	Mixed	Other			
All populations	1,042/15,667	OR	1.89	1.17	1.08	0.98	1.11	1.11	2.04	1.56	0.68	1.05	2.52	1.07	1.009
		P	≈0	≈0	≈0	0.9	0.6	0.4	0.0002	0.01	0.5	0.9	≈0	≈0	≈0
White British	939/13,968	OR	1.96	1.17	1.07	1.13	1.27	1.26					2.62	1.06	1.01
		P	≈0	≈0	≈0	0.4	0.3	0.1					≈0	≈0	≈0

Baseline: sex - female; blood type - B; ethnic background - white.

*≈0 means <10 ^−5^.

### COVID-19 comorbidities

We were interested in the co-occurrence of COVID-19 and comorbidities in individuals who had suffered from severe COVID-19. Therefore, we divided the hospital inpatient diagnosis records into before and after the COVID-19 pandemic using the date 16 March 2020, when COVID-19 testing commenced in the UK. We performed association testing for each comorbidity using logistic regression models and adjusted COVID-19 severity (if the patient received critical care treatments) by sex, age, BMI, SES, smoking and aged care status. Tables 11 and 12 list the top ten associated diseases with severe COVID-19 before and after 16 March 2020. respectively. From Table 12, we found that the common co-occurrence associated with COVID-19 are pneumonia, respiratory diseases, renal failure, metabolic disorders, hypertensive diseases, heart disease and other bacterial diseases. People who have ever had mental disorders, influenza and pneumonia, renal failure, respiratory diseases, bacterial, viral, or other infections, malignant neoplasms of lymphoid, haematopoietic and related tissue, or other blood diseases, tend to have severe symptoms after being infected by SARS-CoV-2.

**Table 11.** The top 10 comorbidities associated with COVID-19 severity before COVID-19 testing in the UK. We divided the hospital inpatient diagnosis records into before and after the COVID-19 pandemic using the date 16 March 2020, when COVID-19 testing commenced. We performed association testing for each comorbidity using logistic regression models and adjusted COVID-19 severity (if the patient received critical care treatments) by sex, age, body mass index, socioeconomic status, smoking and aged care status. To show the comorbidities in individuals who had suffered from severe COVID-19, we ranked the p-values before 16 March 2020 and listed the top 10 comorbidities.

ICD10 code	Diseases	Before 16 March 2020					After 16 March 2020				
		OR	2.50%	97.50%	P-value	Rank	OR	2.50%	97.50%	P-value	Rank
F00-F09	Organic, including symptomatic, mental disorders	2.33	1.86	2.89	4.76E-14	1	2.33	1.88	2.88	5.94E-15	15
J09-J18	Influenza and pneumonia	2.03	1.67	2.46	5.05E-13	2	11.34	9.69	13.28	4.62E-201	1
N17-N19	Renal failure	1.93	1.60	2.30	1.15E-12	3	4.02	3.38	4.78	9.57E-56	4
J95-J99	Other diseases of the respiratory system	2.24	1.77	2.83	1.09E-11	4	13.32	10.94	16.24	1.59E-145	3
J80-J84	Other respiratory diseases principally affecting the interstitium	3.89	2.60	5.78	2.55E-11	5	12.05	8.00	18.28	2.90E-32	6
C81-C96	Malignant neoplasms, stated or presumed to be primary, of lymphoid, haematopoietic and related tissue	3.60	2.44	5.23	4.67E-11	6	5.92	3.93	8.87	8.82E-18	13
B95-B98	Bacterial, viral and other infectious agents	1.93	1.58	2.34	4.81E-11	7	9.01	7.71	10.54	1.22E-166	2
J20-J22	Other acute lower respiratory infections	2.07	1.66	2.58	1.09E-10	8	2.62	1.75	3.87	1.90E-06	31
A30-A49	Other bacterial diseases	2.21	1.72	2.82	3.22E-10	9	3.54	2.71	4.59	5.49E-21	10
D70-D77	Other diseases of blood and blood-forming organs	3.07	2.12	4.39	1.49E-09	10	4.22	2.81	6.29	2.44E-12	18

**Table 12.** The top 10 comorbidities associated with COVID-19 severity after COVID-19 testing in the UK. We divided the hospital inpatient diagnosis records into before and after the COVID-19 pandemic using the date 16 March 2020, when COVID-19 testing commenced. We performed association testing for each comorbidity using logistic regression models and adjusted COVID-19 severity (if the patient received critical care treatments) by sex, age, body mass index, socioeconomic status, smoking and aged care status. To show the top 10 co-occurrence of COVID-19, we ranked the p-values after 16 March 2020 and listed the top 10 comorbidities.

ICD10 code	Diseases	Before 16 March 2020					After 16 March 2020				
		OR	2.50%	97.50%	P-value	Rank	OR	2.50%	97.50%	P-value	Rank
J09-J18	Influenza and pneumonia	2.03	1.67	2.46	5.05E-13	2	11.34	9.69	13.28	4.62E-201	1
B95-B98	Bacterial, viral and other infectious agents	1.93	1.58	2.34	4.81E-11	7	9.01	7.71	10.54	1.22E-166	2
J95-J99	Other diseases of the respiratory system	2.24	1.77	2.83	1.09E-11	4	13.32	10.94	16.24	1.59E-145	3
N17-N19	Renal failure	1.93	1.60	2.30	1.15E-12	3	4.02	3.38	4.78	9.57E-56	4
E70-E90	Metabolic disorders	1.43	1.23	1.66	1.76E-06	19	3.38	2.87	3.97	4.48E-49	5
J80-J84	Other respiratory diseases principally affecting the interstitium	3.89	2.60	5.78	2.55E-11	5	12.05	8.00	18.28	2.90E-32	6
I10-I15	Hypertensive diseases	1.23	1.06	1.43	0.007	50	2.40	2.06	2.80	8.37E-29	7
I30-I52	Other forms of heart disease	1.51	1.29	1.76	2.25E-07	15	2.56	2.16	3.02	8.45E-28	8
J40-J47	Chronic lower respiratory diseases	1.45	1.23	1.70	8.18E-06	22	2.68	2.22	3.21	1.45E-25	9
A30-A49	Other bacterial diseases	2.21	1.72	2.82	3.22E-10	9	3.54	2.71	4.59	5.49E-21	10

### APOE e4

**Figure 6.**
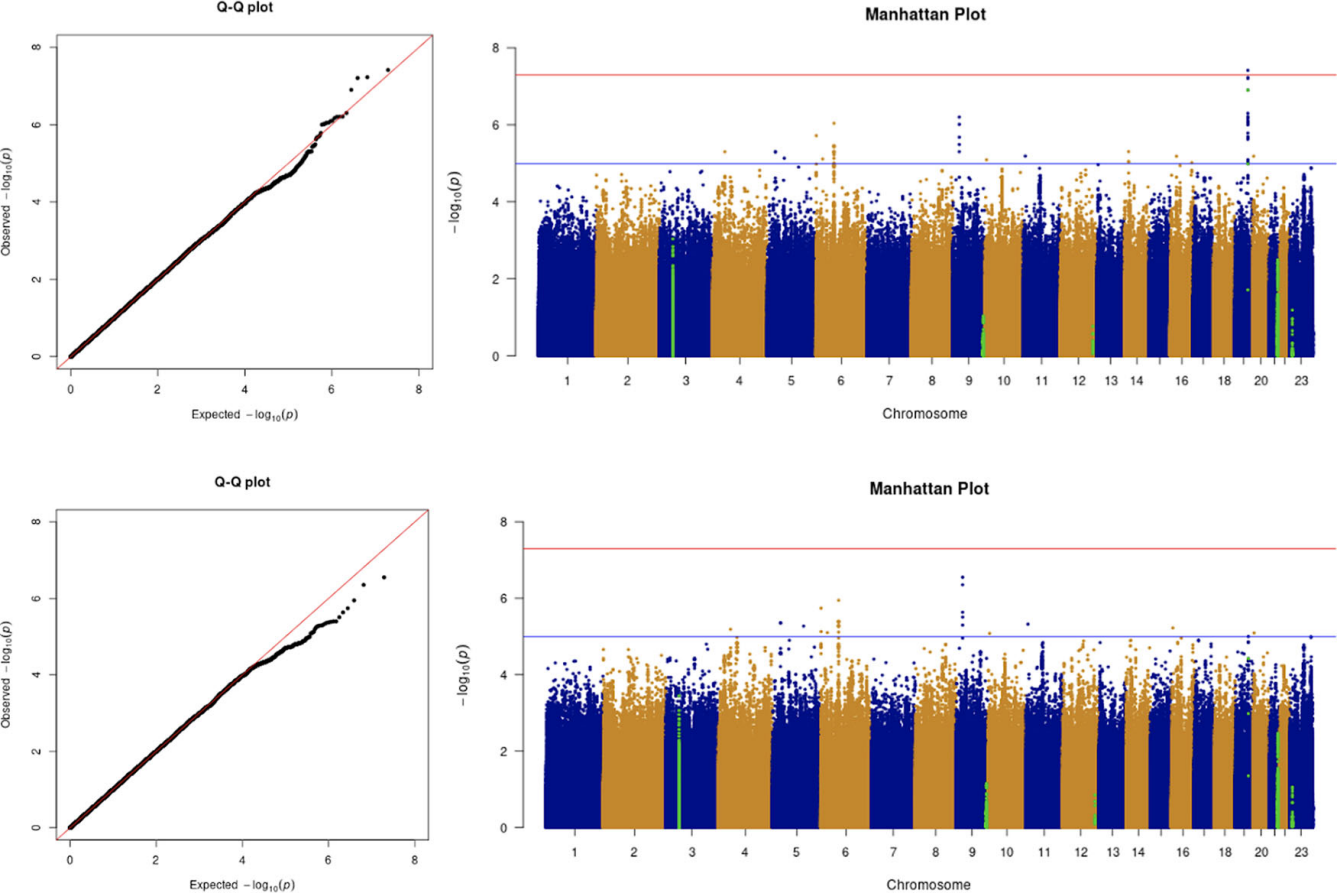
COVID-19 susceptibility GWAS tests with and without aged care status covariate adjustment. a. COVID-19 susceptibility GWAS without care home status covariate adjustment. The model we used is: susceptibility ~ age + sex + BMI + PC1-20 + array + SNP. b. COVID-19 susceptibility GWAS with care home status covariate adjustment. The model we used is: susceptibility ~ age + sex + BMI + PC1-20 + array **+** inAgedCare + SNP. The *APOE e4* signal was genome-wide significant without aged care status but was gone after aged care status adjustment, suggesting that this finding is not robust and may be due to ascertainment bias.

Several publications have reported that the *APOE e4* genotype is associated with COVID-19 susceptibility and severity ( Numbers and Brodaty 2021; Kuo *et al*., 2020a, 2020b). *APOE e4* is a known risk factor for dementia, which has been replicated many times ( Liu *et al*., 2013; Safieh, Korczyn, and Michaelson 2019; Emrani *et al*., 2020). One explanation for people with *APOE e4* being at higher risk of COVID-19 could be due to a higher risk of exposure, as these individuals are more likely to reside in care homes, which have suffered from high rates of infections. This is particularly likely to be the case in UKBB, where 47% of participants are older than 70 years old. To test this hypothesis, we performed GWAS tests with and without aged care status. The *APOE e4* signal was genome-wide significant without aged care status but was gone after aged care status adjustment ( Figure 6), suggesting that this finding is not robust and may be due to ascertainment bias.

## Use cases

To demonstrate the functionality and utility of UKB.COVID19, we present a basic tutorial for using UKB.COVID19. Due to the restriction of using UKBB data, we illustrate the use cases using simulated data. The SAIGE GWAS script example can be found in Github: https://github.com/bahlolab/UKB.COVID19/tree/main/inst/GWAS.

### Basic usage

***Generating a covariate file.*** The ***risk_factor*** function in UKB.COVID19 can be used to generate a covariate file with established risk factors and risk factors of interest by specifying the field code in UKBB main data.

library (UKB.COVID19)

covar <- risk_factor (ukb.data=covid_example("sim_ukb.tab.gz"),

      ABO.data=covid_example("sim_covid19_misc.txt.gz"),

      hesin.file=covid_example("sim_hesin.txt.gz"),

      res.eng=covid_example("sim_result_england.txt.gz"))

head (covar)

#> ID sex age bmi ethnic other.ppl black asian mixed white SES smoke blood_group O AB B A inAgedCare

#> 1 1 1 74 39.0947 1001 0 0 0 0 1 5.43719 0.000 AO 0 0 0 1 0

#> 2 2 1 58 25.3177 1001 0 0 0 0 1 2.10787 0.000 AO 0 0 0 1 0

#> 3 3 0 51 32.2349 1002 0 0 0 0 1 7.36321 25.625 AO 0 0 0 1 0

#> 4 4 0 56 21.7955 1001 0 0 0 0 1 5.62047 0.000 AO 0 0 0 1 0

#> 6 6 1 67 25.9823 1001 0 0 0 0 1 3.90245 0.000 OO 1 0 0 0 0

***Generating COVID-19 susceptibility phenotype file with risk factors.*** In the output file, columns “pos.neg” and “pos.ppl” are the susceptibility phenotypes, which denote 1) UKBB participants with COVID-19 positive versus negative results 2) and participants with positive results versus all the other participants.

phe <- makePhenotypes (ukb.data=covid_example("sim_ukb.tab.gz"),

      res.eng=covid_example("sim_result_england.txt.gz"),

      death.file=covid_example("sim_death.txt.gz"),

      death.cause.file=covid_example("sim_death_cause.txt.gz"),

      hesin.file=covid_example("sim_hesin.txt.gz"),

      hesin_diag.file=covid_example("sim_hesin_diag.txt.gz"),

      hesin_oper.file=covid_example("sim_hesin_oper.txt.gz"),

      hesin_critical.file=covid_example("sim_hesin_critical.txt.gz"),

      code.file=covid_example("coding240.txt.gz"),

      pheno.type = "susceptibility"),

#> [1] "965 participants got tested until 2021-04-05."

#> [1] "218 participants got positive test results until 2021-04-05."

#> [1] "There are 21 deaths with COVID-19. 20 of them primary death cause is COVID-19."

#> [1] "50 patients admitted to hospital were diagnosed as COVID-19 until 2021-04-05."

#> [1] "32 patients' primary diagnosis is COVID-19."

#> [1] "1 patients in hospitalisation with COVID-19 diagnosis but show negative in the result file. Modified their test results."

#> [1] "There are 219 COVID-19 patients identified. 32 individuals are admitted to hospital. 3 had been in ICU. 1 had been in advanced ICU."

#> [1] "Outputting file: ~/UKB.COVID19/extdata/results/phenotype.txt"

head (phe)

#> ID pos.neg pos.ppl

#> 1 1  1  1

#> 2 2  0  0

#> 3 3  0  0

#> 4 4  0  0

#> 5 5  0  0

#> 6 6  0  0

***Performing association tests.*** The ***log_cov*** function performs association tests using logistic regressions. This is an example of association tests between COVID-19 susceptibility and three risk factors: sex, age and BMI.

log_cov(pheno=phe, covariates=covar, phe.name="pos.neg", cov.name=c("sex", "age", "bmi"))

#>   Estimate   OR 2.5 % 97.5 %   p

#> (Intercept) -0.16475743 0.8480994 0.1954585 3.6381032 0.824991899

#> sex1  0.04207813 1.0429760 0.7644672 1.4215535 0.790121307

#> age  -0.03080456 0.9696651 0.9519878 0.9876397 0.001009957

#> bmi  0.03625193 1.0369170 1.0076088 1.0667564 0.012568486

***Generating a comorbidity summary file.*** The ***comorbidity_summary*** function scans all the hospitalisation records with a given time period and generates a text file. The following example is to generate a comorbidity summary file that includes all the primary and secondary diagnoses in the hospital inpatient data after 16 March 2020.

comorb <- comorbidity_summary (ukb.data=covid_example("sim_ukb.tab.gz"),

      hesin.file=covid_example("sim_hesin.txt.gz"),

      hesin_diag.file=covid_example("sim_hesin_diag.txt.gz"),

      ICD10.file=covid_example("ICD10.coding19.txt.gz"),

      primary = FALSE,

      Date.start = "16/03/2020")

comorb[1:6,1:10]

#> ID A00-A09 A15-A19 A20-A28 A30-A49 A50-A64 A65-A69 A70-A74 A75-A79 A80-A89

#> 1 1 1 0 0 1 0 0 0 0 0

#> 2 10 0 0 0 0 0 0 0 0 0

#> 3 100 0 0 0 0 0 0 0 0 0

#> 4 1000 0 0 0 0 0 0 0 0 0

#> 5 101 0 0 0 0 0 0 0 0 0

#> 6 102 0 0 0 0 0 0 0 0 0

***Performing association tests between COVID-19 phenotype and comorbidities.*** This is an example of association tests between COVID-19 susceptibility and all comorbidities. It shows NAs when fitted probabilities numerically 0 or 1 occurred in the logistic regression models.

comorb.asso <- comorbidity_asso (pheno=phe,

      covariates=covar,

      cormorbidity=comorb,

      population="white",

      cov.name=c("sex","age","bmi","SES","smoke","inAgedCare"),

      phe.name="pos.neg",

      ICD10.file=covid_example("ICD10.coding19.txt.gz"))

head (comorb.asso, 4)

#>      ICD10 Estimate  OR 2.5% 97.5% p

#> A00-A09 A00-A09 Intestinal infectious diseases 0.4722864 1.603657 0.756784 3.240022 0.199664372

#> A15-A19 A15-A19 Tuberculosis  NA  NA  NA  NA  NA

#> A20-A28 A20-A28 Certain zoonotic bacterial diseases  NA  NA  NA  NA  NA

#> A30-A49 A30-A49 Other bacterial diseases 1.2246077 3.402831 1.633209 6.978689 0.000873076

## Discussion

We developed an R package that can reproducibly analyse and produce input files for GWAS studies for COVID-19 traits, using the UKBB resource.

The R package can be easily applied to the frequently updated UKBB COVID-19 datasets, facilitating rapid analyses. By applying the R package to data released in April 2021, we found that age, BMI, SES and smoking are positively associated with COVID-19 susceptibility, severity and mortality. Males are at a higher risk of COVID-19 infection than females. People residing in aged care homes were also at higher risk, potentially because they have other pre-existing conditions, and may also have a higher chance of exposure to SARS-CoV-2. By performing GWAS, we replicated previous findings ( Pairo-Castineira *et al*., 2021; Zeberg and Pääbo, 2020; “Genomewide Association Study of Severe Covid-19 with Respiratory Failure”, 2020; Host Genetics Initiative, 2021) that the locus 3p21.31 is associated with COVID-19 susceptibility and severity.

The COVID-19 Host Genetics Initiative brings together the human genetics community to generate, share, and analyse data to learn the genetic determinants of COVID-19 susceptibility, severity, and related outcomes. They have been performing large-scale meta-analyses using existing biobanks, including UKBB, and periodically provide updated releases of their results, making available genome-wide summary statistics, and providing an online browser for exploring the latest results ( https://app.covid19hg.org/). We primarily advocate the use of these resources for exploring genetic associations with COVID-19 susceptibility and severity. However, we anticipate our R package will enable researchers to undertake more bespoke genetic analyses, using the most up to date UKBB COVID-19 data, to meet the aim of their studies. Such analyses may include adjusting for non-genetic risk factors or comorbidities, to explore mediators, polygenic risk score analyses, or Mendelian Randomisation studies.

Long COVID, also known as post-acute sequelae of SARS-CoV-2 infection, refers to a range of symptoms that persist for weeks or months after the acute phase of COVID-19 has resolved. These symptoms can include fatigue, shortness of breath, cognitive dysfunction, and various other systemic issues, significantly impacting the quality of life of affected individuals. The UKB.COVID19 package provides multiple functions to facilitate long COVID analysis. For instance, the ‘comorbidity_summary’ and ‘comorbidity_asso’ functions can be used to summarise potential long COVID symptoms and assess their associations with risk factors, such as age, sex and certain pre-existing conditions. Furthermore, researchers can focus on subsets of participants reporting persistent symptoms consistent with long COVID to investigate genetic risk factors using GWAS. These analyses hold promise for uncovering the biological underpinnings of long COVID and identifying potential therapeutic targets to alleviate its impact.

There are several limitations of UKBB COVID-19 data. First, UKBB is not a nationally or worldwide representative sample. The majority of participants are of white British ethnicity. UKBB participants were more likely to be older, to be female, and to live in less socioeconomically deprived areas than nonparticipants. Compared with the general population, participants were less likely to be obese, to smoke, and to drink alcohol daily and had fewer self-reported health conditions ( Fry *et al*., 2017). Initiatives such as OpenSafely ( Williamson *et al*., 2020), have aimed to examine risk factors for COVID-19 disease in an unascertained UK population, via electronic health records. These data, however, are not presently available for use by the wider research community, due to the possibility of re-identification of individuals. The recent OpenSafely flagship paper examined health records of over 17 million individuals in England, of whom 10,926 had a COVID-19 related death, and found that male sex, greater age and deprivation, and non-white ethnicities were major clinical risk factors for mortality. Despite the ascertainment of the UKBB, it is reassuring that these established risk factors are also associated with COVID-19 outcomes in this cohort.

Second, the UKBB COVID-19 dataset evolved as testing scaled up in line with the national testing strategy and thus COVID-19 data is also subject to ascertainment bias. UK testing was initially largely restricted to healthcare workers, and those individuals with symptoms in hospitals. A positive result in an individual not recorded as a healthcare worker was therefore a reasonable proxy for severe disease early on in the pandemic. Testing capacity subsequently increased to include more community testing under pillar 2 of the national strategy, and as of 27 April 2020, NHS England directed hospitals to test all non-elective patients admitted overnight, including asymptomatic patients. To maximise ascertainment of cases and to evaluate disease severity, SARS-CoV-2 testing data should be used in combination with linked medical records (i.e. hospital inpatient records and death records) as we have implemented in this package. More recently, UKBB has made primary care records available for COVID-19 research. These data not yet utilised by the UKB.COVID19 package, will further improve case identification. Nonetheless, there are likely to be many individuals in the UKBB who contracted COVID-19, in particular those with milder disease, who will not be captured by the available data.

The definition of COVID-19 susceptibility is supposed to be the status of people who get infected or not after exposure to SARS-CoV-2. However, exposure to SARS-CoV-2 is not easy to determine. Furthermore, not everyone has an equal chance of being exposed to SARS-CoV-2 (for example, exposure will vary by occupation), nor does everyone have the same likelihood of being tested, due to testing strategies, as noted above. Such data idiosyncrasies have the potential to distort associations, in observational studies, and also in genetic analyses through population stratification. This issue of ascertainment, or collider bias, in the context of COVID-19, is discussed at length by Griffith *et al*. (2020). Analyses using the UKBB data should therefore be undertaken and interpreted within the context of changing testing capacity, and other limitations regarding phenotype definitions.

We welcome further suggestions and improvements for this R package, which we hope will reduce the barrier to utilising the UKBB data for COVID-19 research.

## Data availability

All the datasets were obtained from UKBB.

To access the UKBB datasets, you need to register as a UKBB researcher ( https://www.ukbiobank.ac.uk/enable-your-research/register). If you are already an approved UKBB researcher with a project underway and wish to receive these datasets for COVID-19 research purposes, you can register to receive these data by logging into the Access Management System (AMS) ( https://bbams.ndph.ox.ac.uk/ams/resApplications).

How to apply for access to UKBB data: https://www.ukbiobank.ac.uk/enable-your-research/apply-for-access. See COVID-19 data ( https://biobank.ndph.ox.ac.uk/showcase/exinfo.cgi?src=COVID19) for registration and access details and Resource 1758 ( https://biobank.ndph.ox.ac.uk/showcase/refer.cgi?id=1758) for further information.

All genome wide significant GWAS hits with gene annotations are shown in Table 7.

## Software availability

UKB.COVID19 can be installed via CRAN using install.packages (“UKB.COVID19”).

UKB.COVID19 is maintained at https://github.com/bahlolab/UKB.COVID19.

Latest UKB.COVID19 source code is available from: https://github.com/bahlolab/UKB.COVID19.

Archived source code at the time of publication: http://doi.org/10.5281/zenodo.5174381 ( Wang *et al*., 2021).

License: MIT ( https://opensource.org/licenses/MIT).

## References

[ref1] Black D : “HEALTH AND DEPRIVATION: Inequality and the North.” *J Royal College General Practitioners.* 1988;38(310):234.

[ref2] Booth A Reed AB Ponzo S : Population Risk Factors for Severe Disease and Mortality in COVID-19: A Global Systematic Review and Meta-Analysis. *PloS One* .2021;16(3):e0247461. 10.1371/journal.pone.0247461 33661992 PMC7932512

[ref3] Bycroft C Freeman C Petkova D : The UK Biobank Resource with Deep Phenotyping and Genomic Data. *Nature.* 2018;562(7726):203–209. 10.1038/s41586-018-0579-z 30305743 PMC6786975

[ref4] Elhabyan A Saja E Ehab S : The Role of Host Genetics in Susceptibility to Severe Viral Infections in Humans and Insights into Host Genetics of Severe COVID-19: A Systematic Review. *Virus Res.* 2020;289(November):198163. 10.1016/j.virusres.2020.198163 32918943 PMC7480444

[ref5] Elliott LT Sharp K Alfaro-Almagro F : Genome-Wide Association Studies of Brain Imaging Phenotypes in UK Biobank. *Nature.* 2018;562(7726):210–216. 10.1038/s41586-018-0571-7 30305740 PMC6786974

[ref6] Emrani S Arain HA DeMarshall C : APOE4 Is Associated with Cognitive and Pathological Heterogeneity in Patients with Alzheimer’s Disease: A Systematic Review. *Alzheimers Res Ther.* 2020. 10.1186/s13195-020-00712-4 33148345 PMC7643479

[ref7] Fry A Littlejohns TJ Sudlow C : Comparison of Sociodemographic and Health-Related Characteristics of UK Biobank Participants With Those of the General Population. *Am J Epidemiol.* 2017;186(9):1026–1034. 10.1093/aje/kwx246 28641372 PMC5860371

[ref8] Genomewide Association Study of Severe Covid-19 with Respiratory Failure. *New Eng J Med.* 2020;383(16):1522–1534. 10.1056/NEJMoa2020283 32558485 PMC7315890

[ref9] Griffith GJ Morris TT Tudball MJ : Collider Bias Undermines Our Understanding of COVID-19 Disease Risk and Severity. *Nat Commun.* 2020;11(1):5749. 10.1038/s41467-020-19478-2 33184277 PMC7665028

[ref10] Hoffmann M Kleine-Weber H Schroeder S : SARS-CoV-2 Cell Entry Depends on ACE2 and TMPRSS2 and Is Blocked by a Clinically Proven Protease Inhibitor. *Cell.* 2020. 10.1016/j.cell.2020.02.052 32142651 PMC7102627

[ref11] Host Genetics Initiative, Covid-19: Mapping the Human Genetic Architecture of COVID-19 by Worldwide Meta-Analysis. *MedRxiv.* 2021; Reference Source

[ref12] Jiang H Promchan K Lin B-R : LZTFL1 Upregulated by All-Trans Retinoic Acid during CD4+ T Cell Activation Enhances IL-5 Production. *J Immunol.* 2016;196(3):1081–1090. 10.4049/jimmunol.1500719 26700766 PMC4724573

[ref13] Kaser A : Genetic Risk of Severe Covid-19. *New England J Med.* 2020. 10.1056/nejme2025501 PMC7583681 33053291

[ref14] Kuo C-L Pilling LC Atkins JL : ApoE e4e4 Genotype and Mortality With COVID-19 in UK Biobank. *The Journals of Gerontology. Series A, Biological Sciences and Medical Sciences.* 2020a;75(9):1801–1803. 10.1093/gerona/glaa169 32623451 PMC7337688

[ref15] Kuo C-L Pilling LC Atkins JL : APOE e4 Genotype Predicts Severe COVID-19 in the UK Biobank Community Cohort. *The Journals of Gerontology. Series A, Biological Sciences and Medical Sciences.* 2020b;75(11):2231–2232. 10.1093/gerona/glaa131 32451547 PMC7314139

[ref16] Liu C-C Liu C-C Kanekiyo T : Apolipoprotein E and Alzheimer Disease: Risk, Mechanisms and Therapy. *Nat Rev. Neurol* 2013;9(2):106–118. 10.1038/nrneurol.2012.263 23296339 PMC3726719

[ref17] Numbers K Brodaty H : The Effects of the COVID-19 Pandemic on People with Dementia. *Nat Rev. Neurol.* 2021;17(2):69–70. 10.1038/s41582-020-00450-z 33408384 PMC7786184

[ref18] Pairo-Castineira E Clohisey S Klaric L : Genetic mechanisms of critical illness in COVID-19. *Nature.* 2021;591:92–98. 10.1038/s41586-020-03065-y 33307546

[ref19] Peckham H Gruijter NM de Raine C : Male Sex Identified by Global COVID-19 Meta-Analysis as a Risk Factor for Death and ITU Admission. *Nat Commun.* 2020;11(1):6317. 10.1038/s41467-020-19741-6 33298944 PMC7726563

[ref20] Pijls BG Jolani S Atherley A : Demographic Risk Factors for COVID-19 Infection, Severity, ICU Admission and Death: A Meta-Analysis of 59 Studies. *BMJ Open.* 2021;11(1):e044640. 10.1136/bmjopen-2020-044640 33431495 PMC7802392

[ref21] Purcell S Neale B Todd-Brown K : PLINK: A Tool Set for Whole-Genome Association and Population-Based Linkage Analyses. *Am J Hum Genet.* 2007;81(3):559–575. 10.1086/519795 17701901 PMC1950838

[ref22] Safieh M Korczyn AD Michaelson DM : ApoE4: An Emerging Therapeutic Target for Alzheimer’s Disease. *BMC Med.* 2019. 10.1186/s12916-019-1299-4 30890171 PMC6425600

[ref23] Seo S Zhang Q Bugge K : A Novel Protein LZTFL1 Regulates Ciliary Trafficking of the BBSome and Smoothened. *PLoS Genet.* 2011;7(11):e1002358. 10.1371/journal.pgen.1002358 22072986 PMC3207910

[ref24] Wang L Jackson VE Fearnley LG : UKB.COVID19: an R package for UK Biobank COVID-19 data processing and analysis. *Zenodo.* 2021. 10.5281/zenodo.5174381 PMC11347911 39193262

[ref25] Williamson EJ Walker AJ Bhaskaran K : Factors Associated with COVID-19-Related Death Using OpenSAFELY. *Nature.* 2020;584(7821):430–436. 10.1038/s41586-020-2521-4 32640463 PMC7611074

[ref26] Wolff D Nee S Hickey NS : Risk Factors for Covid-19 Severity and Fatality: A Structured Literature Review. *Infection.* 2021;49(1):15–28. 10.1007/s15010-020-01509-1 32860214 PMC7453858

[ref27] Wu Z McGoogan JM : Characteristics of and Important Lessons from the Coronavirus Disease 2019 (COVID-19) Outbreak in China: Summary of a Report of 72 314 Cases from the Chinese Center for Disease Control and Prevention. *JAMA.* 2020;323(13):1239–1242. 10.1001/jama.2020.2648 32091533

[ref28] Zeberg H Pääbo S : The Major Genetic Risk Factor for Severe COVID-19 Is Inherited from Neanderthals. *Nature.* 2020;587(7835):610–612. 10.1038/s41586-020-2818-3 32998156

[ref29] Zhao J Yang Y Huang H : Relationship between the ABO Blood Group and the COVID-19 Susceptibility. *Clinical Infectious Diseases: An Official Publication of the Infectious Diseases Society of America.* August 2020. 10.1093/cid/ciaa1150 PMC7454371 32750119

[ref30] Zhou W Nielsen JB Fritsche LG : Efficiently Controlling for Case-Control Imbalance and Sample Relatedness in Large-Scale Genetic Association Studies. *Nat Genet.* 2018;50(9). 10.1038/s41588-018-0184-y PMC6119127 30104761

[ref31] Zietz M Zucker J Tatonetti NP : Associations between Blood Type and COVID-19 Infection, Intubation, and Death. *Nat Commun.* 2020;11(1):5761. 10.1038/s41467-020-19623-x 33188185 PMC7666188

