# Supplementary material for: UKB.COVID19: an R package for UK Biobank COVID-19 data processing and analysis
Source: F1000Res. 2024 Jul 26;10:830. Originally published 2021 Aug 19. [Version 3] doi: 10.12688/f1000research.55370.3 (PMC11347911; doi:10.12688/f1000research.55370.3)
Supplement: Peer review history

# Reviewer report: Reviewer response for version 3

**DOI:** 10.5256/f1000research.169531.r307380

**Published:** 2024-08-26

**Authors:** Thomas Michael Palmer

**Affiliations:** 1 Population Health Sciences, University of Bristol Medical School, Bristol, UK

I thank the authors for their response and the amendments they have made to the package.

 The package is now back on CRAN. Therefore it again successfully passes R CMD check. So all of my previous comments have essentially been addressed.

 My only remaining minor comments are

- In the vignette I don't think the line: library(here) is required
- In the vignette you might want to set warning=FALSE on a few code chunks because several chunks generate quite alot of warnings which are distracting when reading it  https://cran.r-project.org/web/packages/UKB.COVID19/vignettes/Introduction_to_UKB_COVID19.html

Are the conclusions about the tool and its performance adequately supported by the findings presented in the article?

No

Is the rationale for developing the new software tool clearly explained?

Yes

Is the description of the software tool technically sound?

No

Are sufficient details of the code, methods and analysis (if applicable) provided to allow replication of the software development and its use by others?

Partly

Is sufficient information provided to allow interpretation of the expected output datasets and any results generated using the tool?

Partly

Reviewer Expertise:

Medical statistics, biostatistics, statistics, R programming.

I confirm that I have read this submission and believe that I have an appropriate level of expertise to confirm that it is of an acceptable scientific standard.

# Reviewer report: Reviewer response for version 2

**DOI:** 10.5256/f1000research.133689.r287362

**Published:** 2024-06-12

**Authors:** Edgar Gonzalez-Kozlova

**Affiliations:** 1 Icahn School of Medicine at Mount Sinai, New York, NY, USA

Dear authors, 

 Fantastic job preparing a package to facilitate data retrieval and analysis.

 I would like to see a few additions that can only strengthen the article.

 > Are sufficient details of the code, methods and analysis (if applicable) provided to allow replication of the software development and its use by others?

 Prepare make a vignette available showcasing exactly how you intended the package to be used. While the article describes well the study and package, packages without a vignette are disregarded. 

 > Is sufficient information provided to allow interpretation of the expected output datasets and any results generated using the tool?

 There is no mention of long covid in the article. Long term effects of COVID19 cant be ignored. Please include a discussion and/or status of long covid patients in the article.

 Are the conclusions about the tool and its performance adequately supported by the findings presented in the article?

 A statistics section is missing from the methods section. Every test used in the article should be clearly described and justified in methods.

Are the conclusions about the tool and its performance adequately supported by the findings presented in the article?

Partly

Is the rationale for developing the new software tool clearly explained?

Yes

Is the description of the software tool technically sound?

Yes

Are sufficient details of the code, methods and analysis (if applicable) provided to allow replication of the software development and its use by others?

Partly

Is sufficient information provided to allow interpretation of the expected output datasets and any results generated using the tool?

Partly

Reviewer Expertise:

Computational Biology

I confirm that I have read this submission and believe that I have an appropriate level of expertise to state that I do not consider it to be of an acceptable scientific standard, for reasons outlined above.

# Response

**Published:** 2024-07-23

**Authors:** Longfei Wang

**Affiliations:** WEHI, Australia

**------------------**

**Reviewer Comment:**

**1. Prepare make a vignette available showcasing exactly how you intended the package to be used. While the article describes well the study and package, packages without a vignette are disregarded.**

**Author Response: **We appreciate your suggestion. We have created a vignette and updated the UKB.COVID R package on CRAN ( https://cran.r-project.org/web/packages/UKB.COVID19/vignettes/Introduction_to_UKB_COVID19.html).

**------------------**

**Reviewer Comment:**

**2. There is no mention of long covid in the article. Long term effects of COVID19 cant be ignored. Please include a discussion and/or status of long covid patients in the article.**

**Author Response: **Thank you for your suggestion. We have added a discussion of long COVID and provided relevant functions in UKB.COVID19.

 Long COVID, also known as post-acute sequelae of SARS-CoV-2 infection, refers to a range of symptoms that persist for weeks or months after the acute phase of COVID-19 has resolved. These symptoms can include fatigue, shortness of breath, cognitive dysfunction, and various other systemic issues, significantly impacting the quality of life of affected individuals. The UKB.COVID19 package provides multiple functions to facilitate long COVID analysis. For instance, the ‘comorbidity_summary’ and ‘comorbidity_asso’ functions can be used to summarise potential long COVID symptoms and assess their associations with risk factors, such as age, sex and certain pre-existing conditions. Furthermore, researchers can focus on subsets of participants reporting persistent symptoms consistent with long COVID to investigate genetic risk factors using GWAS. These analyses hold promise for uncovering the biological underpinnings of long COVID and identifying potential therapeutic targets to alleviate its impact.

**------------------**

**Reviewer Comment:**

**3. A statistics section is missing from the methods section. Every test used in the article should be clearly described and justified in methods.**

**Author Response: **We added a statistics section in the methods section.

**Statistical analysis**

 To assess the associations between non-genetic risk factors and COVID-19 phenotypes (including susceptibility, severity, and mortality), we employed multivariable logistic regression models using the ‘glm’ function from the R package stats. Each model adjusted for covariates such as age, sex, and BMI. The tested risk factors included socioeconomic status (SES), smoking status, blood type, ethnic background, and residence in aged care facilities. The logistic regression model for each risk factor was specified as follows: logit(COVID-19 phenotype) ~ risk factor + age + sex + BMI. Comorbidity associations were analyzed using similar multivariable logistic regression models, with COVID-19 phenotypes modeled as: logit(COVID-19 phenotype) ~ comorbidity category + age + sex + BMI + SES + smoking status + aged care status. Odds ratios (ORs) with 95% confidence intervals (CIs) were reported, and p-values were calculated to determine the significance of the associations.

 To identify genetic variants associated with COVID-19 phenotypes, we performed GWASs using the SAIGE software. Principal component analysis (PCA) was performed to account for population stratification, and the first 20 principal components (PCs) were included as covariates in the analysis. Additionally, we adjusted for age, sex, BMI, SES, smoking status, residence in aged care facilities and genotypic array in the regression models. The association between each SNP and the phenotypes was tested using a logistic regression model, as follows: logit(COVID-19 phenotype) ~ SNP + age + sex + BMI + SES + smoking status + aged care status + genotypic array + PC1-20. To account for multiple testing, the Bonferroni correction was applied. Loci reaching the genome-wide significance threshold (p < 5x10 ^-8^) were considered significant. Manhattan plots and quantile-quantile (QQ) plots were generated to visualize the results using R package ggplot2. All analyses were carried out using R (version 4.0.5).

**------------------**

# Reviewer report: Reviewer response for version 1

**DOI:** 10.5256/f1000research.58938.r126903

**Published:** 2022-04-22

**Authors:** Annalisa De Silvestri, Virginia Valeria

**Affiliations:** 1 Servizio di Epidemiologia Clinica e Biostatistica Direzione Scientifica, Fondazione IRCCS Policlinico san Matteo, Pavia, Italy; 2 Scientific Direction, IRCCS Policlinico San Matteo Foundation, Pavia, Italy

Authors developed a potentially useful R-package tool to analyze data from the UKBB COVID-19 database, which summarises COVID-19 test results, and performs association tests between COVID-19 susceptibility/severity and potential risk factors such as age, sex, blood type, comorbidities and generates input files for GWAS.

 The rationale is well explained, sufficient details of the code, methods, and analysis are provided, outputs are well described and conclusions are sound and appropriate.

 However, some minor points should be considered:

- It is not clear how comorbidities are retrieved, classified (at which level of ICD-10), and analysed
- Authors should discuss how they choose to classify severity (the distinction between critical care and advanced critical care for example) and why they choose to include all Covid patients (for example severity 2-3 vs 0-1 instead of severity 2-3 vs 1). Why not consider it as an ordinal variable?
- Authors should specify if they consider mortality due to Covid or with Covid

Are the conclusions about the tool and its performance adequately supported by the findings presented in the article?

Yes

Is the rationale for developing the new software tool clearly explained?

Yes

Is the description of the software tool technically sound?

Partly

Are sufficient details of the code, methods and analysis (if applicable) provided to allow replication of the software development and its use by others?

Yes

Is sufficient information provided to allow interpretation of the expected output datasets and any results generated using the tool?

Yes

Reviewer Expertise:

biostatistics

We confirm that we have read this submission and believe that we have an appropriate level of expertise to confirm that it is of an acceptable scientific standard.

# Response

**Published:** 2022-05-02

**Authors:** Longfei Wang

**Affiliations:** WEHI, Australia

**1. It is not clear how comorbidities are retrieved, classified (at which level of ICD-10), and analysed.**

 We added a new table (Table 4) to show how the comorbidities are classified. We modified the following sentences:

*Comorbidity categories are generated using the block categories in the ICD10 code, which is shown in the second column in Table 4. We include ICD10 chapters 1–14 and 17 and exclude several chapters such as pregnancy, childbirth, and consequences of external causes etc. For instance, the first category is “A00-A09”, representing intestinal infectious diseases. During a period restricted by the start and end dates, cases are defined as any participants who were diagnosed as any subclasses under the block A00-A09 in the hospital inpatient diagnosis data. In this way, 164 binary variables are generated and each of them represents a comorbidity category.*

 Therefore, we can test the association between each comorbidity category and the selected COVID-19 phenotype using logistic regression models. We modified the sentence as follows:

*The comorbidity.asso function performs association tests between each comorbidity category and the selected phenotype using logistic regression models and adjusts the tested phenotype with covariates, which can be set using the argument “cov.name”.*

****

**2. Authors should discuss how they choose to classify severity (the distinction between critical care and advanced critical care for example) and why they choose to include all Covid patients (for example severity 2-3 vs 0-1 instead of severity 2-3 vs 1). Why not consider it as an ordinal variable?**

 Thanks for the suggestion. We re-wrote the following paragraph:

 Based on the World Health Organization (WHO) ordinal scale for clinical improvement, we classify severity into four levels. These levels are defined as 1) hospitalisation: individuals admitted to hospital with their primary diagnosis recorded as COVID-19. 2) critical care level 2: individuals required basic treatment in a critical care unit, such as non-invasive ventilation and continuous positive airway pressure, and with their primary diagnosis recorded as COVID-19. 3) critical care level 3: individuals required advanced treatment in a critical care unit, such as invasive ventilation and temporary tracheostomy, and with their primary diagnosis recorded as COVID-19. 4) mortality: individuals died due to COVID-19. The critical care information was summarised from the HESIN_CRITICAL table and the HESIN_OPER table. The critical care level 2 cases are the COVID-19 patients who required at least one “Critical care level 2 days” in the HESIN_CRIRICAL table or received basic respiratory support, such as, E85.2 non-invasive ventilation NEC, in the HESIN_OPER table. The critical care level 3 cases are defined as the COVID-19 patients who required at least one “Critical care level 3 days” in the HESIN_CRIRICAL table or received advanced respiratory support, such as, E85.1 invasive ventilation, in the HESIN_OPER table. The commonly used GWAS tools, such as SAIGE and PLINK, do not support ordinal categorical phenotypes. Therefore, we converted this ordinal variable into four binary variables named “hospitalisation”, “critical care”, “advanced critical care” and “mortality” (Table 2). However, users can get the ordinal variable by simply summing the four binary variables. We assume that participants who were tested COVID-19 positive but did not admit to hospital had no or mild symptoms and hence classified them as controls in severity phenotypes.

****

**3. Authors should specify if they consider mortality due to Covid or with Covid**

 Sorry for the unclearness. We defined the mortality case as mortality due to Covid. In the article, we wrote:

*For mortality, we include all individuals who received at least one positive test result and define those whose primary cause of death is recorded as being due to COVID-19 as cases.*

 To make it clearer, we corrected the definition of mortality in Table 2 from “1 = death with COVID-19” to “1 = death due to COVID-19”.

# Reviewer report: Reviewer response for version 1

**DOI:** 10.5256/f1000research.58938.r100445

**Published:** 2021-12-02

**Authors:** Thomas Michael Palmer

**Affiliations:** 1 Population Health Sciences, University of Bristol Medical School, Bristol, UK

Before I review this R package properly there are some basic fixes to the GitHub repository version which require attention.

1. The package has an unusual history. Two versions have been released on CRAN however as I can see from the website it was “Archived on 2021-10-06 as email to the maintainer was undeliverable”. So I recommend that the authors contact CRAN to get the package unarchived.
2. The CRAN archive shows versions 0.1.0 and 0.1.1, however the GitHub repo shows version 0.1.0 in its DESCRIPTION file. The repo should have the latest version in it.
3. Whilst the versions listed on CRAN 0.1.0 and 0.1.1 must have been CRAN compliant, otherwise they would not have been allowed on CRAN, unfortunately the code in the GitHub is no longer CRAN compliant and a simple running of R CMD check on the code in the repo gives 2 R CMD check errors and 1 note. These R CMD check errors should be fixed and the R CMD check note should also be fixed by adding the relevant entries to the .Rbuildignore file.
4. The script in the tests/testthat folder does not use any of the testthat functions as it should. This should be improved or removed.
5. Personally I find the name of the package unusual, I don’t prefer full-stops/periods in package names.
6. Returned objects from the functions could be defined under one of the R's class systems, e.g., S3.

Are the conclusions about the tool and its performance adequately supported by the findings presented in the article?

No

Is the rationale for developing the new software tool clearly explained?

Yes

Is the description of the software tool technically sound?

No

Are sufficient details of the code, methods and analysis (if applicable) provided to allow replication of the software development and its use by others?

Partly

Is sufficient information provided to allow interpretation of the expected output datasets and any results generated using the tool?

Partly

Reviewer Expertise:

Medical Statistics / Biostatistics

I confirm that I have read this submission and believe that I have an appropriate level of expertise to state that I do not consider it to be of an acceptable scientific standard, for reasons outlined above.

# Response

**Published:** 2022-01-04

**Authors:** Longfei Wang

**Affiliations:** WEHI, Australia

*1.The package has an unusual history. Two versions have been released on CRAN however as I can see from the website it was “Archived on 2021-10-06 as email to the maintainer was undeliverable”. So I recommend that the authors contact CRAN to get the package unarchived.*

 My apologies that I was not aware that the package had been archived. I have contacted the CRAN team. They replied that a CRAN team member tried to contact me and the email has got a bounced message notification. However, my email address is correct and has not been changed since I submitted the package. I have resubmitted the package with an increased version number and with minor changes according to your suggestions. It has been unarchived

 ( https://cran.r-project.org/web/packages/UKB.COVID19/index.html).

*2. The CRAN archive shows versions 0.1.0 and 0.1.1, however the GitHub repo shows version 0.1.0 in its DESCRIPTION file. The repo should have the latest version in it.*

 My apologies that the package on GitHub was out-of-date. I have updated the latest version in GitHub.

*3. Whilst the versions listed on CRAN 0.1.0 and 0.1.1 must have been CRAN compliant, otherwise they would not have been allowed on CRAN, unfortunately the code in the GitHub is no longer CRAN compliant and a simple running of R CMD check on the code in the repo gives 2 R CMD check errors and 1 note. These R CMD check errors should be fixed and the R CMD check note should also be fixed by adding the relevant entries to the .Rbuildignore file.*

 I have updated the latest version in GitHub and double checked it with R CMD check. There’s no errors, warnings, or notes from the R CMD check now.  

*4. The script in the tests/testthat folder does not use any of the testthat functions as it should. This should be improved or removed.*

 Thanks for your suggestion. I have improved the scripts in the tests/testthat folder with proper testthat functions.

*5. Personally I find the name of the package unusual, I don’t prefer full-stops/periods in package names.*

 Thanks for your suggestion. The package has been on CRAN for a while. People may have included the package in their scripts. These scripts will break if I change the name of the package. And it may be hard for everyone to find the renamed package. So I decided to keep the name and will definitely use proper names for the packages I build in the future.

*6. Returned objects from the functions could be defined under one of the R's class systems, e.g., S3.*

 Thanks for your suggestion. I have defined the returned objects under the S3 class system.
